# Self‐Assembly of Organic Semiconductors on Strained Graphene under Strain‐Induced Pseudo‐Electric Fields

**DOI:** 10.1002/advs.202400598

**Published:** 2024-03-13

**Authors:** Jinhyun Hwang, Jisang Park, Jinhyeok Choi, Taeksang Lee, Hyo Chan Lee, Kilwon Cho

**Affiliations:** ^1^ Department of Chemical Engineering Pohang University of Science and Technology Pohang 37673 Republic of Korea; ^2^ Department of Mechanical Engineering Myongji University Yongin 17058 Republic of Korea; ^3^ Department of Chemical Engineering Myongji University Yongin 17058 Republic of Korea

**Keywords:** graphene, growth template, organic semiconductor, pseudo‐electric field, strain engineering

## Abstract

Graphene is used as a growth template for van der Waals epitaxy of organic semiconductor (OSC) thin films. During the synthesis and transfer of chemical‐vapor‐deposited graphene on a target substrate, local inhomogeneities in the graphene—in particular, a nonuniform strain field in the graphene template—can easily form, causing poor morphology and crystallinity of the OSC thin films. Moreover, a strain field in graphene introduces a pseudo‐electric field in the graphene. Here, the study investigates how the strain and strain‐induced pseudo‐electric field of a graphene template affect the self‐assembly of π‐conjugated organic molecules on it. Periodically strained graphene templates are fabricated by transferring graphene onto an array of nanospheres and then analyzed the growth and nucleation behavior of C_60_ thin films on the strained graphene templates. Both experiments and a numerical simulation demonstrated that strained graphene reduced the desorption energy between the graphene and the C_60_ molecules and thereby suppressed both nucleation and growth of the C_60_. A mechanism is proposed in which the strain‐induced pseudo‐electric field in graphene modulates the binding energy of organic molecules on the graphene.

## Graphical abstract

The self‐assembly of C_60_ molecules on a strained graphene surface is investigated. Tensile strain in graphene induces a shift in its work function, inhibiting electron transfer from graphene to C_60_. Consequently, the reduced Coulomb interaction leads to the desorption of C_60_ molecules from the strained graphene surface, thereby suppressing C_60_ nucleation.

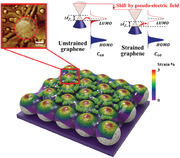

## 1 Introduction

Graphene, a 2D carbon allotrope, has a π‐conjugated system that connects the 2*p_z_* orbitals of adjacent carbon atoms. Because of this π‐conjugated system, graphene has been used as a growth template for organic semiconductor (OSC) thin films.^[^ ^1^, ^2^ ^]^ The π‐conjugated graphene surface shows strong van der Waals interaction with π‐conjugated molecules.^[^ ^3^ ^]^ Therefore, when graphene is used as a growth template, π‐conjugated molecules self‐align to maximize π–π interactions with the template; the resultant OSC thin films therefore have a large grain size and high crystallinity. The graphene template also offers wide tunability of the crystal structure, microstructure, and surface morphology of OSC thin films because various surface characteristics of graphene, such as their doping level, roughness, and surface energy, can be easily modified.^[^ ^4^, ^5^, ^6^ ^]^ In addition, vacuum deposition of OSC thin films directly onto a graphene template yields graphene/organic hybrid materials that have an atomically clean interface.^[^ ^7^ ^]^ As a result, high‐performance optoelectronic devices that exploit the advantages of graphene/organic hybrid materials have been fabricated using graphene templates.^[^ ^8^, ^9^, ^10^ ^]^

Because of its thinness and mechanical robustness,^[^ ^11^ ^]^ graphene can withstand large mechanical strain without fracture;^[^ ^12^ ^]^ thus, strain engineering has been widely used to modulate the chemical reactivity,^[^ ^13^ ^]^ plasmonic properties,^[^ ^14^ ^]^ and quantum transport of charge carriers^[^ ^15^, ^16^, ^17^, ^18^ ^]^ in graphene. In particular, the strain field in graphene generates pseudo‐magnetic fields or pseudo‐electric fields and the charge carriers in the strained graphene behave as they would under real magnetic or electric fields.^[^ ^17^ ^]^

Under the tight‐binding model, strains in graphene that modify the relative orientation of its atoms result in the modulation of nearest‐neighbor and next‐nearest‐neighbor hopping and spacing between the 2*p_z_* orbitals of the carbon atoms. The change in nearest‐neighbor hopping and spacing leads to pseudo‐vector potentials (and, thus, to pseudo‐magnetic fields), whereas the change in next‐nearest‐neighbor hopping and spacing induces a pseudo‐scalar potential (and, thus, pseudo‐electric fields) in graphene.^[^ ^18^, ^19^ ^]^ Pseudo‐magnetic fields in graphene have recently become an important research topic because the strain‐induced pseudo‐magnetic fields can reach as high as 800 T; they therefore provide a basis for the experimental study of extremely high magnetic fields in graphene as well as a promising platform for scalable valleytronic devices based on graphene.^[^ ^20^ ^]^

Pseudo‐potentials in graphene affect its electronic structure in the same manner as real electric scalar potentials; thus, strain‐induced pseudo‐electric fields can affect the interaction between graphene and OSC molecules in the same manner as real electric fields.^[^ ^21^ ^]^ The effects of strain‐induced pseudo‐potentials in graphene would be important when the graphene is used as a growth template because graphene on a substrate is easily subjected to large mechanical deformations. Thermal cycling during the synthesis and transfer of graphene induces mechanical strain in graphene because of a mismatch between the thermal expansion coefficients of the graphene and the substrate.^[^ ^22^ ^]^ In addition, surface roughness of the substrate can introduce mechanical strain into graphene;^[^ ^23^ ^]^ thus, pseudo‐potentials in graphene can be a key factor that determines the self‐assembly of OSC molecules on a graphene template. Despite their possible importance, the effects of strain‐induced pseudo‐potentials in a graphene template on the self‐assembly of OSC molecules have not yet been investigated.

Here, we demonstrate that strain‐induced pseudo‐electric fields in a graphene template affect the nucleation and growth of OSC thin films. To induce pseudo‐electric fields in a graphene template in a controlled manner, we transferred graphene onto an array of silica nanospheres (NS‐array) with a diameter of ≈200 nm.^[^ ^24^ ^]^ We then used atomic force microscopy (AFM) to investigate the nucleation and growth behavior of C_60_ thin films on the graphene/NS‐array templates and also conducted a finite‐difference method (FDM) numerical simulation of C_60_ ad‐molecules on the graphene/NS‐array templates. These investigations revealed that pseudo‐electric fields in graphene govern the probability of electron transfer between the graphene and C_60_ ad‐molecules. The pseudo‐electric fields thereby change the interaction of the C_60_ ad‐molecules with the graphene and, thus, the nucleation and growth behavior of the C_60_ thin films. Our findings open new avenues for controlling the electronic properties of graphene/organic interfaces and other 2D materials/organic interfaces via strain engineering and may pave the way for the development of novel optoelectronic devices.

## 2 Results and Discussion

### 2.1 Nucleation and Growth of C_60_ Thin Film on Strained Graphene Template

**Figure 1:**
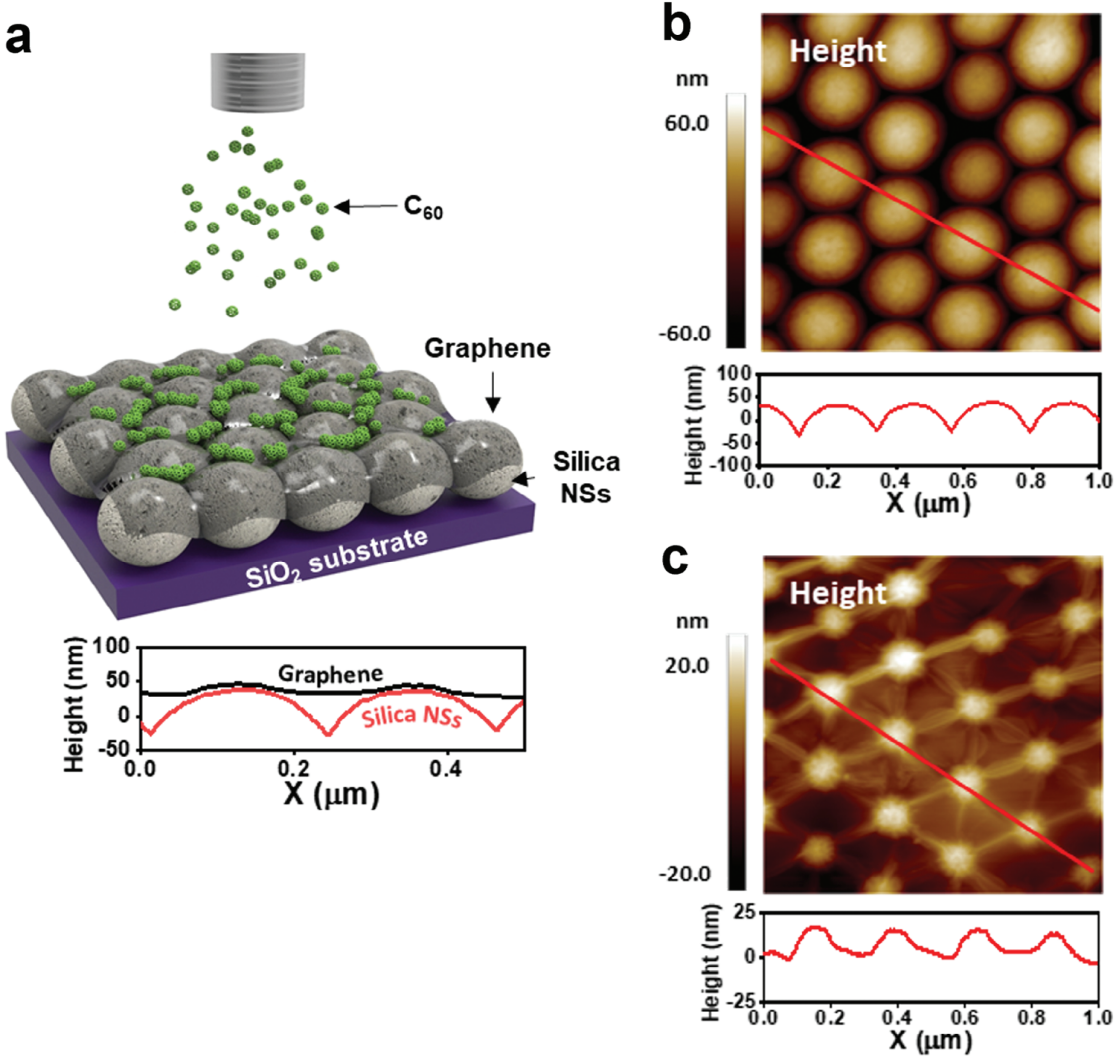
C_60_ growth on strained graphene. a) Scheme showing vapor deposition of C_60_ on graphene/NS‐array(G/NS‐array). AFM image b) before transfer of graphene onto the NS‐array and c) after transfer of graphene onto the NS‐array.

In our experimental system (**Figure** **1**a), silica nanospheres (NSs) with a diameter of 200 ± 10 nm were dispersed at a concentration of 10 mg mL^−1^ in deionized (DI) water and spin‐coated onto 300 nm thick SiO_2_/Si. The NSs aggregated spontaneously to form hexagonal close‐packed NS arrays (Figure 1b). The NS cluster size ranged from a few microns to tens of microns. On the NS‐array, chemical‐vapor‐deposited graphene (CVD graphene) was transferred via the wet transfer method (see also Figure S1, Supporting Information).^[^ ^25^ ^]^ To eliminate undesirable polymer residues or trapped water molecules, which can be introduced during the wet transfer, the samples of graphene on an NS‐array (G/NS‐array) were annealed under H_2_ (Figure S2, Supporting Information).

The relationship between the topography and strain distribution of G/NS‐arrays has been well investigated.^[^ ^24^, ^26^ ^]^ Experimental and simulation results have demonstrated that the apex of NSs is subjected to tensile strain, whereas the graphene in a freestanding region of a G/NS‐array is subjected to nearly zero strain.^[^ ^24^ ^]^ The areal ratio between the strained region and the unstrained region is determined by the balance between the strain energy of graphene and the adhesion energy between NSs and graphene at the apex. The adhesion energy between graphene and NSs compensates for the strain energy of graphene because of the deformation of graphene on NSs. Lastly, a C_60_ thin film was vapor‐deposited onto the G/NS‐array substrate under ultra‐high vacuum (UHV, ≈10^−8^ Torr) to verify the nucleation behavior of the OSC thin film.

The AFM height image (Figure 1b) of a bare NS‐array shows that the height of the apex from the valley is ≈60 nm. Compared with the height of the bare NS‐array, the height difference between the apex of the G/NS‐array and the free‐standing region of graphene is only ≈20 nm (Figure 1c), which is a sign of partial conformal contact of graphene on the NS array; that is, some regions of graphene are in direct contact with NSs and other regions are free‐standing. Here, we define the free‐standing regions of graphene as the areas not attached to the NSs and surrounded by wrinkles connecting the apexes of the G/NS‐array. To further investigate the structure of the G/NS‐array, we analyzed typical cross‐sectional profiles of the NS and G/NS templates (Figure S3, Supporting Information). Approximately 1/10 of the area of the graphene regions was measured to have a radius of curvature smaller than or almost identical to that of a bare NS. We interpreted these results as indicating that 10% of the graphene surface was conformally in contact with NSs and the rest was free‐standing. The curvature of the conformal contact regions of the graphene was on the order of 10^−3^ nm^−1^, and the free‐standing graphene was nearly flat.

**Figure 2:**
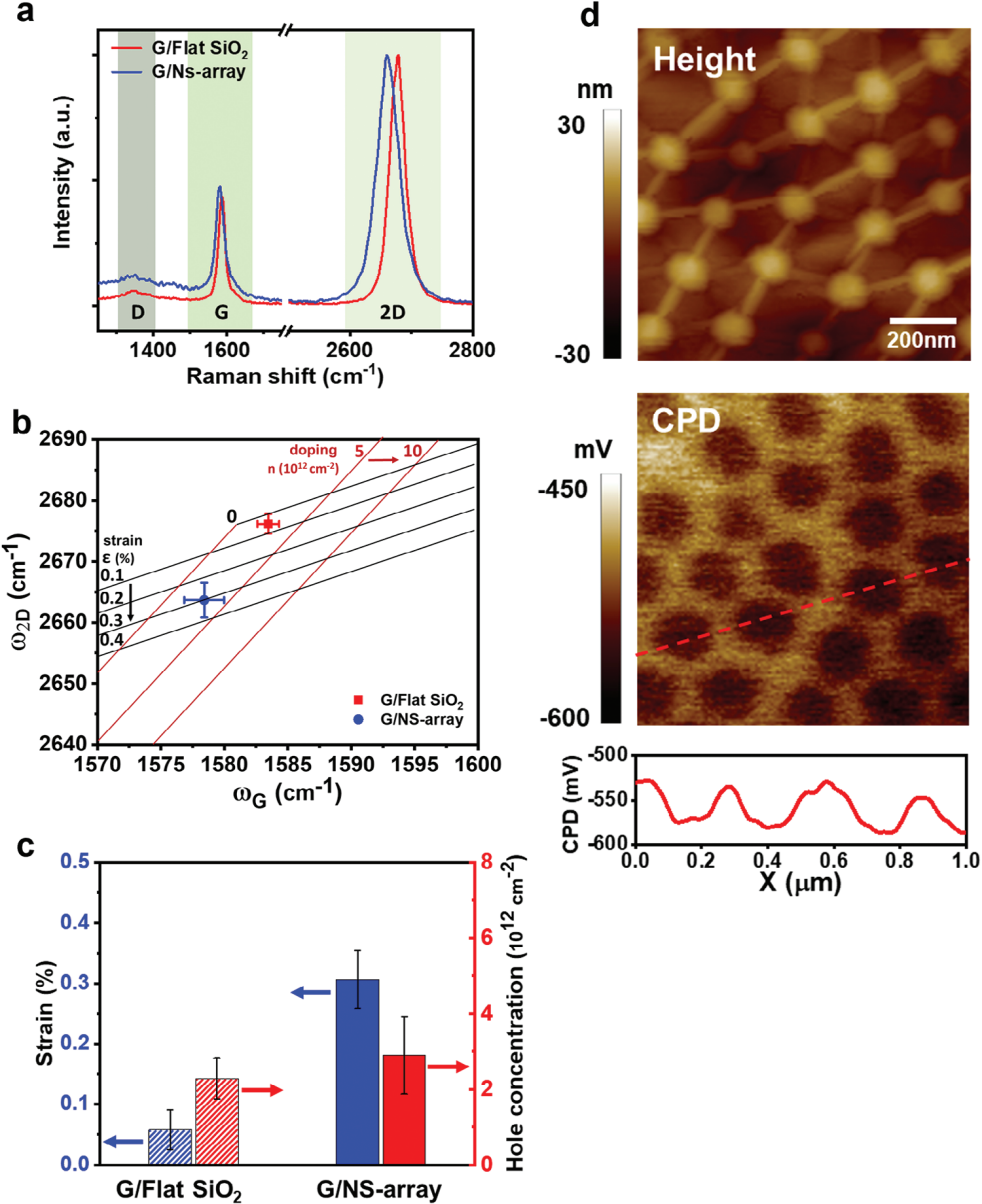
Strain/doping quantification by Raman mapping. a) Raman single spectrum of G/Flat SiO_2_ and G/NS‐array. b) Strain and doping‐induced shift of the G and 2D peaks. Average value and standard deviation of 2D, G peak plotted on (ω_G._, ω_2D_) map for G/Flat SiO_2_ (red square) and G/NS‐array (blue circle). c) Histogram of calculated average tensile strain and average hole concentration of G/Flat SiO_2_ (dashed line bar) and G/NS‐array (solid line bar); blue bar: tensile strain; red bar: average hole concentration. d) Height image (upper) and contact potential difference (CPD) image (lower) of G/NS‐array.

The G and 2D Raman peaks of graphene can be shifted by strain and doping.^[^ ^27^ ^]^ The Raman single spectrum (**Figure** **2**a) shows a clear redshift of the G and 2D peaks of graphene on the NS array compared with the corresponding peaks of graphene on flat SiO_2_ (G/Flat SiO_2_); this difference implies that the graphene on the NS array is subjected to substantial tensile strain.^[^ ^28^, ^29^ ^]^ To qualitatively measure the strain and doping level of the G/NS‐array, we extracted the strain and doping levels of graphene from (ω_G_, ω_2D_) data using a previously reported method.^[^ ^24^, ^27^ ^]^ The G and 2D peaks corresponding to twenty points on each G/NS‐array and G/Flat SiO_2_ sample were collected from different locations by Raman spectroscopy and subsequently plotted on a (ω_G_, ω_2D_) map (Figure 2b). The estimated average tensile strain was 0.3% on the G/NS‐array sample but only ≈0.05% on the G/Flat SiO_2_ sample; this difference demonstrates that the use of the NS‐array successfully controlled the strain in graphene (Figure 2c). The laser spot size was ≈500 nm in diameter; thus, the tensile strain measured by Raman spectroscopy is the spatially averaged tensile strain in graphene. Because the strain in free‐standing graphene regions is relatively small, the actual strain applied in graphene on the apex of NS should be much larger than the strain estimated from Raman spectroscopic analysis. Assuming that free‐standing graphene is nearly strain‐free,^[^ ^30^ ^]^ the graphene areas that conformally contact NS have an average tensile strain of ≈3.0% on a G/NS array sample (see details in Figure S3, Supporting Information). The defect density of the G/NS‐array samples were estimated to be ≈4 × 1010 cm^−2^ (0.001% defect content), as obtained from the intensity ratio between the D and G peaks.^[^ ^31^ ^]^ Therefore, the effects of defects in graphene on the strain distribution would be inconsequential.^[^ ^32^ ^]^

The estimated average hole‐doping levels were 2.9 × 1012 cm^−2^ in the G/NS‐array sample and 2.3 × 1012 cm^−2^ in the G/Flat SiO_2_ sample. The difference between them is within the range of error (Figure 2c) and is therefore not significant. The negligible difference in the doping level between the partially supported graphene (G/NS‐array) and the fully supported graphene (G/Flat SiO_2_) implies that the hole doping level in the apex regions of the G/NS array is similar to that in the free‐standing regions; this inference is consistent with the results of a previous report.^[^ ^24^ ^]^

The Raman spectroscopic analysis provides quantitative values for the strain in the G/NS‐array; however, only spatially averaged levels can be estimated. To obtain information on the spatial distribution of strain, we conducted Kelvin probe force microscopy (KPFM) measurements using n‐doped Si tips (Figure 2d). The KPFM method measures the local work function of graphene by measuring the contact potential difference (CPD), which is given by CPD  = (φ_tip_ − φ_sample_)/*e*  where φ_tip_ is the work function of the n‐doped Si tips (≈4.1 eV), φ_sample_ is the work function of graphene, and *e* is the elementary charge. In our experimental setup, a more negative CPD value indicates a deeper work function of graphene. The spatial resolution of the KPFM measurement is comparable to or greater than the diameter of a single silica nanosphere because of the cantilever of the AFM tip. Therefore, the local work function measured at a point represents the spatially averaged work function around the point. Nonetheless, the KPFM measurements qualitatively provided the relative local work function of graphene.

Because the doping levels of the graphene at the apex regions and in the free‐standing regions are similar, the difference in the local work function should be attributable to strain‐induced pseudo‐electric fields. As a result, a comparison of the height and CPD images of a G/NS‐array clearly shows that the work function of graphene is deeper at the apex regions than in the free‐standing regions (Figure 2d). The resolution of the Kelvin probe force microscope enables a measurement of the CPD of wrinkles (Figure S4, Supporting Information); however, we did not observe any CPD contrast between the wrinkles and the free‐standing regions of the G/NS‐array, indicating that negligible strains were applied in the wrinkles. Although the KPFM study does not allow for a quantitative estimation of the actual strain levels in the G/NS‐array,^[^ ^33^ ^]^ it provides clear evidence that the strain distribution is consistent with our assumptions and with previously reported simulation results that tensile strain is localized at the apex regions.^[^ ^30^ ^]^

**Figure 3:**
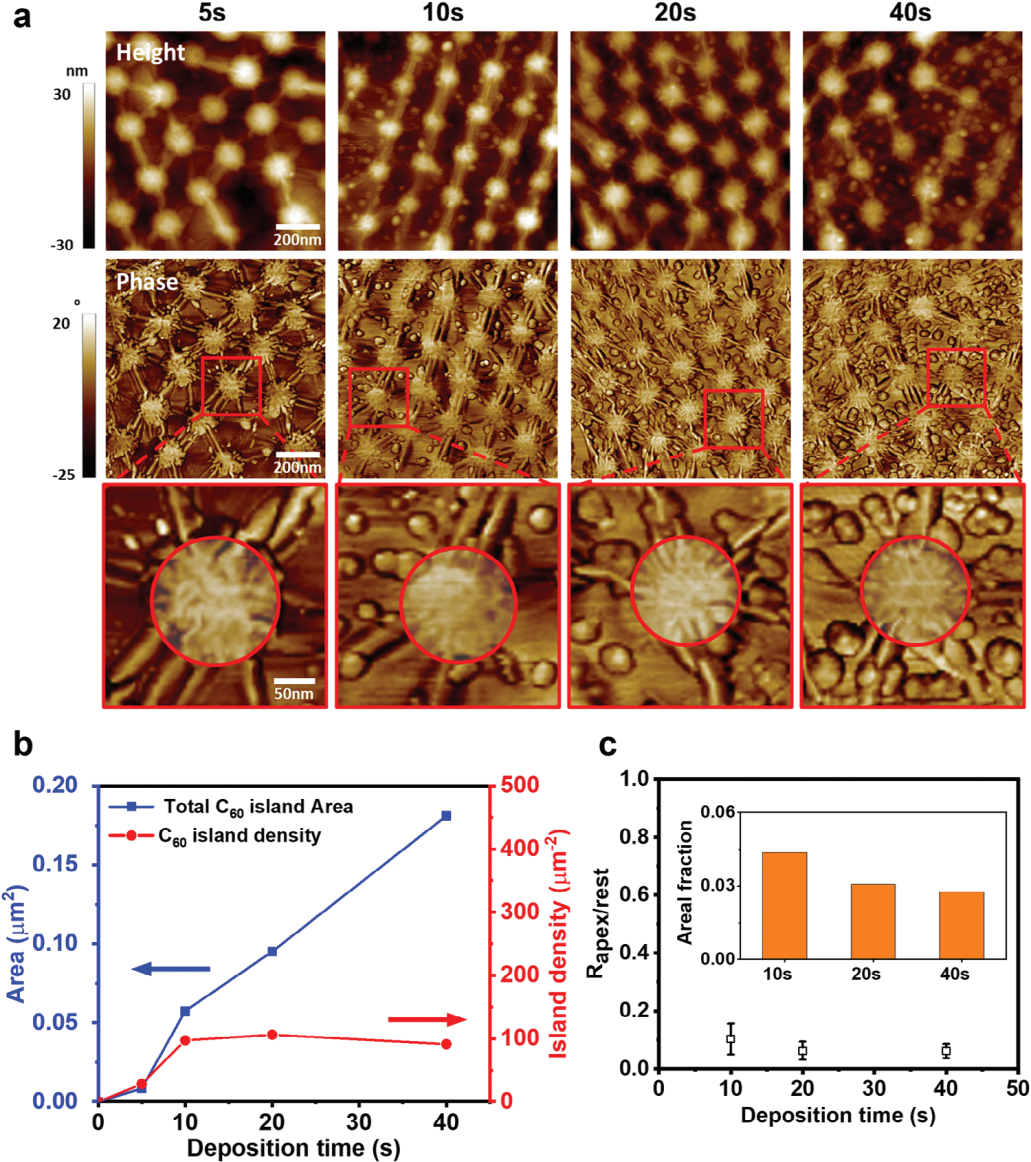
Nucleation of C_60_ islands on G/NS‐array. a) AFM height and phase images of C_60_ at deposition time of 5, 10, 20, or 40 s. Image with red circled region indicating graphene with conformal contact on apex of NS was magnified from red squares in the phase image. b) C_60_ island area (blue line) and island number density (red line) according to deposition time of C_60_. c) Ratio of the coverage of C_60_ islands at the apex regions to that of the rest of the regions in the G/NS‐array template (*R*_apex/rest_) as a function of the deposition time. Inset: fraction of the C_60_ islands area at the apex regions in the G/NS‐array templates to the total C_60_ islands area as a function of the deposition time.

The AFM images in **Figure** **3**a show C_60_ thin films on a G/NS‐array during the early stage of nucleation (nominal thickness < 1 ML; see Figures S5–S7, Supporting Information). Because of the wavy surface morphology of the G/NS‐array, clear identification of C_60_ islands in the height images was difficult. However, C_60_ islands were clearly observed in the phase images; we therefore used them in our analysis of the nucleation and growth behavior of the C_60_ thin films.

When C_60_ was deposited for 5 s at a rate of 0.1 Å s^−1^, only a few C_60_ islands formed and the total area of the C_60_ islands was negligible (Figure 3b). Rapid nucleation of a C_60_ thin film began after 5 s of deposition, and the growth of C_60_ islands without additional nucleation was observed after 10 s of deposition. An incubation time (≈5 s) for C_60_ nucleation can be observed if the critical nucleus size of C_60_ is substantially larger than one molecule or if C_60_ ad‐molecules are desorbed from the graphene.^[^ ^34^, ^35^, ^36^, ^37^ ^]^ In general, the critical nucleus size of C_60_ is considered to be one molecule for various substrates;^[^ ^38^ ^]^ thus, the incubation time indicates that C_60_ ad‐molecules readily desorbed from the graphene before nucleation at room temperature.

Because the C_60_ islands were thicker than a C_60_ monolayer, we concluded that the C_60_ grew in Volmer–Weber growth mode rather than in layer‐by‐layer growth mode. The Volmer–Weber growth mode might be caused by the transfer of electrons from the graphene to the C_60_ ad‐molecules or C_60_ islands. The charge transfer generates Coulombic repulsion between the C_60_ ad‐molecules and C_60_ islands, then prevents the C_60_ ad‐molecules from attaching to pre‐existing C_60_ islands, resulting in Volmer–Weber growth.^[^ ^5^ ^]^

Notably, C_60_ islands were specifically located on the free‐standing regions of graphene, away from the apex regions. This result is interesting because C_60_ islands formed easily on the top of the NSs when graphene was absent (Figure S8, Supporting Information). *R*_apex/rest_ is defined as the ratio of the coverage of C_60_ islands at the apex regions to that at other regions in the G/NS‐array template. As *R*_apex/rest_ approaches zero, it indicates the nucleation and growth of C_60_ is suppressed on the apex regions. The areal fraction, that is, the area of the C_60_ islands at the apex regions in the G/NS‐array templates to the total area of the C_60_ islands, was 4.3%, and *R*_apex/rest_ was 0.10 when C_60_ was deposited for 10 s (Figure 3c). The areal fraction and *R*_apex/rest_ decreased to 2.7% and 0.06, respectively, when the deposition time was increased from 10 to 40 s, indicating that the C_60_ islands grew faster in the direction toward the free‐standing regions than in the direction toward the apex regions (see Figure S9, Supporting Information). Notably, only a few C_60_ islands were observed at the tips of the wrinkles. The possible strain at the tips of the wrinkles may cause the desorption of C_60_ molecules, or the high curvature of wrinkles may introduce unfavorable deformation energy in C_60_ thin films.^[^ ^39^ ^]^

### 2.2 Effects of Graphene Doping and Strain on C_60_ Nucleation Behavior

**Figure 4:**
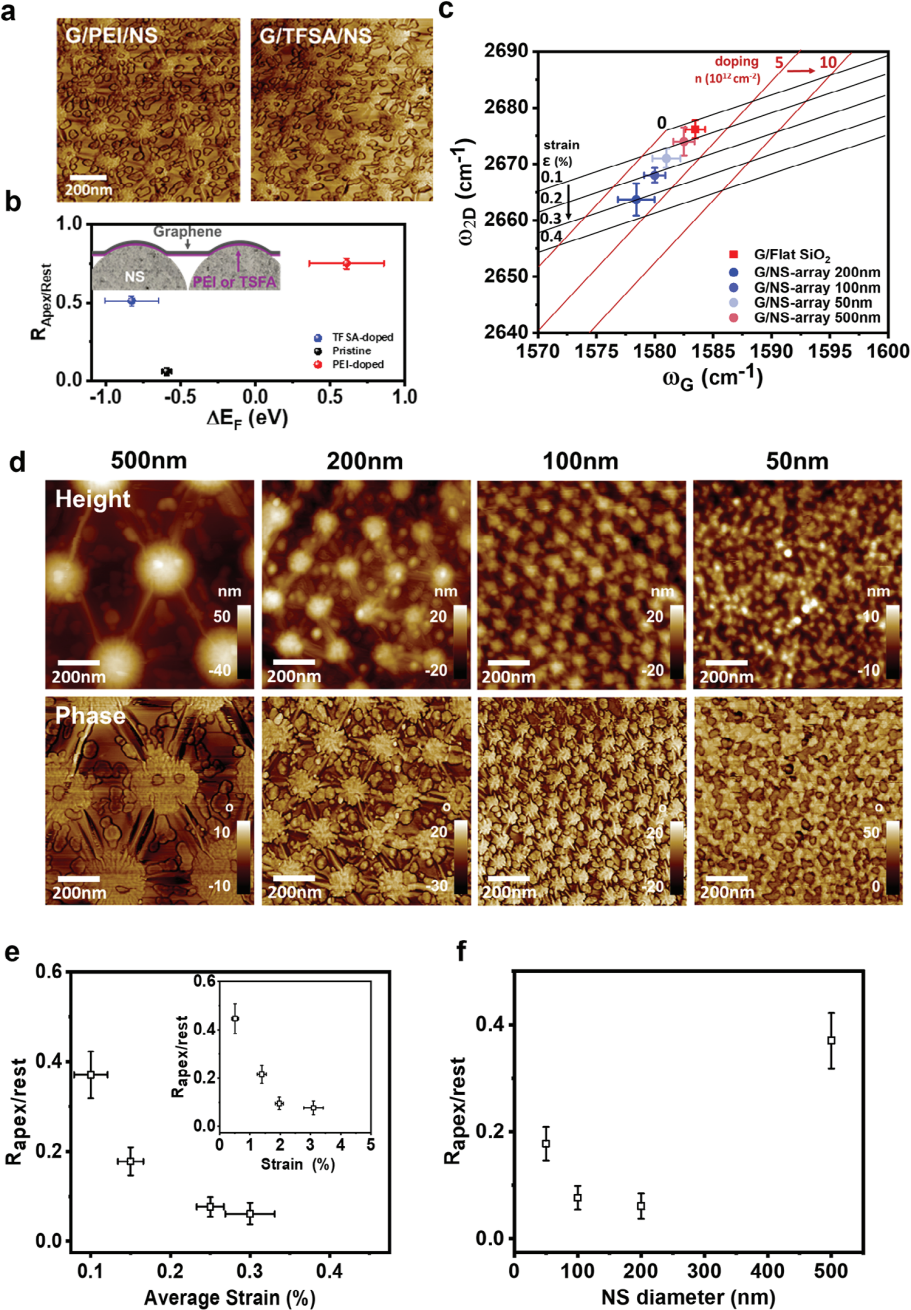
Nucleation of C_60_ islands on G/NS‐arrays with different strains and doping levels. a) AFM phase images of C_60_ on p‐doped (G/TFSA/NS) and n‐doped (G/PEI/NS) samples. b) *R*_apex/rest_ as a function of ΔE_F_. ΔE_F_ is the Fermi level shift with respect to E_D_ due to doping. c) Average value and standard deviation of 2D and G peaks plotted on a (ω_G._, ω_2D_) map for G/Flat SiO_2_ (red square) and G/NS‐arrays with different NS diameters (50–500 nm; 50 nm: gray circle, 100 nm: light blue circle, 200 nm: blue circle, and 500 nm: light red circle). d) AFM height and phase images of G/NS‐arrays with 50–500 nm diameter NSs. e) *R*_apex/rest_ as a function of the average strain of G/NS‐arrays estimated from Figure 4c (Inset: *R*_apex/rest _as a function of the graphene strain on the apex regions). f) *R*_apex/rest _ as a function of the NS diameter.

We modulated the doping level of graphene in G/NS‐array templates (**Figure** **4**a,b). To this end, the bottom‐side doping method was employed.^[^ ^40^ ^]^ The graphene doping level was estimated using Raman spectroscopy analysis and the fabrication of field‐effect transistors (Figure S10, Supporting Information). When bis(trifluoromethanesulfonyl)amide (TFSA) was used as the dopant, graphene became more p‐doped, with a hole concentration of 4–5 × 1012 cm^−2^. The use of poly(ethylene imine) (PEI) induced n‐doping of the G/NS‐array templates, and the electron concentration was 2–3 × 1012 cm^−2^. The C_60_ thin film was then deposited as 0.4 ML onto both the TFSA‐doped G/NS‐array (G/TFSA/NS‐array) and PEI‐doped G/NS‐array (G/PEI/NS‐array). Figure 4a shows that the nucleation density of C_60_ on the apex regions of both the G/PEI/NS‐ and G/TFSA/NS‐array samples noticeably increased. The *R*_apex/rest_ values for the G/PEI/NS‐ and G/TFSA/NS‐array templates were estimated to be 0.75 and 0.51, respectively. The *R*_apex/rest_ value for G/TFSA/NS‐array template was slightly lower than that for the G/PEI/NS template. Because both the G/PEI/NS‐ and G/TFSA/NS‐array templates exhibited the same surface morphology as the undoped G/NS‐array templates, the observed variation in *R*_apex/rest_ due to graphene doping implies that fluctuations in the height or curvature of graphene induced by the underlying nanospheres have minimal effects on the nucleation and growth behaviors of C_60_ on the G/NS‐array templates. The nucleation density of C_60_ on graphene templates is reportedly determined by the degree of electron transfer from graphene to the C_60_ ad molecules.^[^ ^4^ ^]^ Therefore, the dependence of the growth behavior of C_60_, especially regarding *R*_apex/rest_, on the doping level of graphene strongly indicates that the charge transfer between graphene and C_60_ plays a significant role in determining the behavior of the C_60_ growth and *R*_apex/rest_.

Subsequently, the strain field on the apex region was modulated by transferring graphene onto NS arrays of varying diameters (50, 100, 200, and 500 nm; Figure S11, Supporting Information). The graphene strain and doping levels were extracted from the (ω_G_, ω_2D_) data (Figure 4c). The doping level of all G/NS‐array templates was ≈2.5 × 1012 cm^−2^ regardless of the NS size. However, the average strain of the G/NS arrays changed with the changing NS diameters. Assuming that free‐standing graphene was nearly strain‐free, the apex strains for the 500, 50, 100, and 200 nm NS arrays were estimated to be 0.51, 1.39, 1.97, and 3.1%, respectively (see details in Figure S3, Supporting Information).

A C_60_ thin film was then deposited onto the strain‐controlled G/NS‐arrays, and the dependence of *R*_apex/rest_ on the graphene strain was analyzed using AFM (Figure 4d). *R*_apex/rest_ gradually decreased from 0.37 to 0.06 with an increasing average strain (Figure 4e) or local strain at the apex regions (Figure 4e, inset) of the G/NS‐array templates (Figure 4e).

Notably, no distinct correlation between *R*_apex/rest_ and the graphene curvature was observed (Figure 4f). To understand this observation, density functional theory (DFT) calculations were performed on the C_60_/graphene systems. Increasing the curvature to 0.026 nm^−1^ (radius of curvature = 37.8 nm) very slightly decreased the van der Waals interaction energy of C_60_ on graphene. DFT calculations were also performed to calculate the effect of mechanical strain in graphene on the van der Waals interactions of C_60_ molecules with graphene (see details in Table S1 and Figure S12, Supporting Information). In summary, the clear dependence of *R*_apex/rest_ on the strain of the G/NS‐array templates in the apex regions implies that the strain in the apex regions affects the charge transfer between graphene and C_60_.

The proposed mechanism was corroborated by observing the nucleation behavior of other OSC materials (pentacene and 1,4,5,8‐naphthalenetetracarboxylic dianhydride (NTCDA)) on graphene/NS‐array templates (see further details in Figure S13, Supporting Information). Pentacene, in the absence of charge transfer because of its high lowest unoccupied molecular orbital (LUMO) level, demonstrated a relatively high *R*_apex/rest_ of ≈1.47. NTCDA has a LUMO energy level of 4.0 eV, which induces electron transfer from graphene to the OSC. Similar to the C_60_ case, the corresponding *R*_apex/rest_ for NTCDA was measured to be 0.09.

### 2.3 Growth Mechanism of C_60_ Thin film Under Strained‐Induced Pseudo‐Electric Fields of Graphene

**Figure 5:**
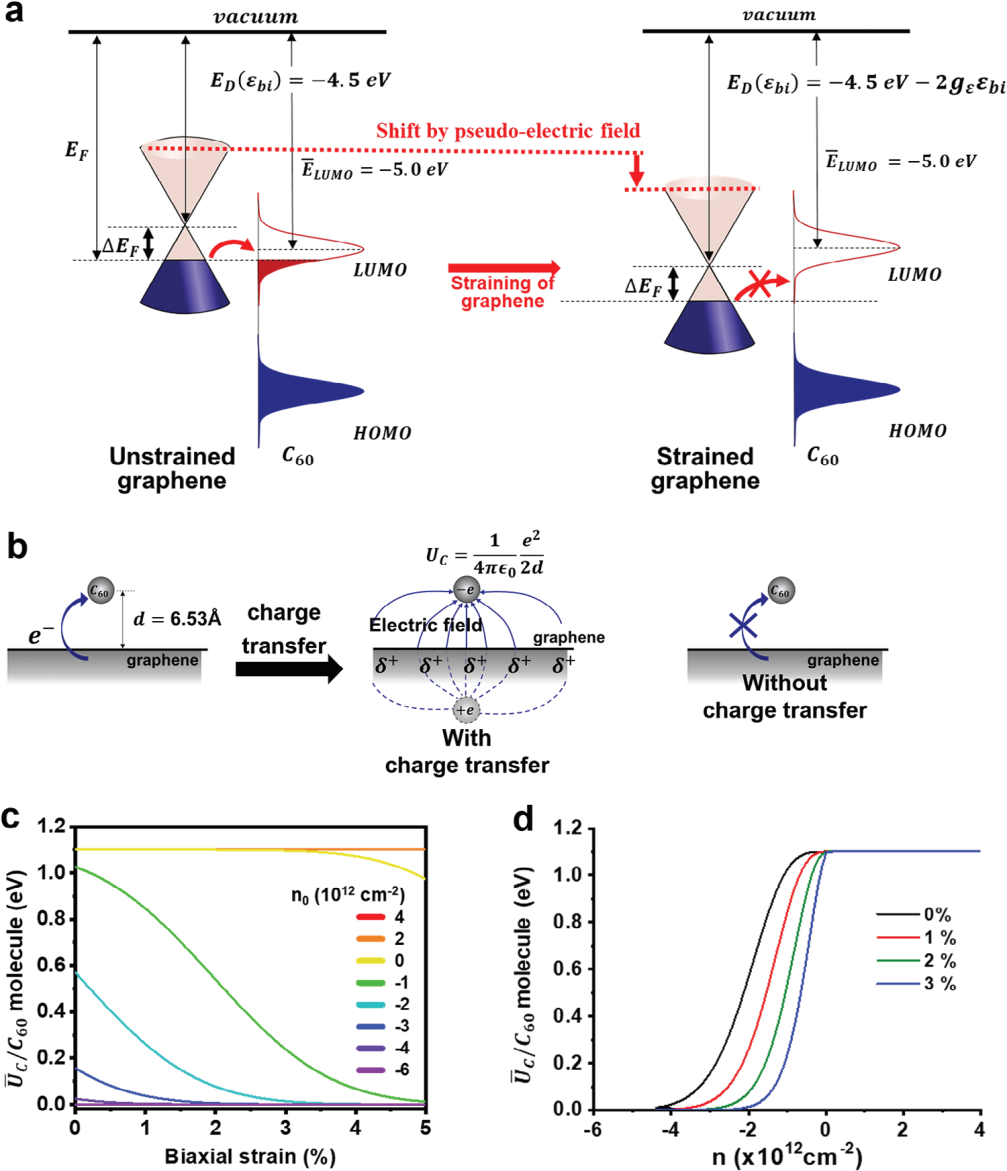
Effect of strain applied on graphene on interfacial state between C_60_ and graphene. a) Energy‐band diagrams of graphene/C_60_ with no strain applied (left) and with strain applied to graphene (right); red arrow from dashed line: work‐function shift by strain‐induced pseudo‐electric field. b) Schematic illustrations of C_60_/graphene interaction of van der Waals energy and additional Coulombic energy by image charge method. c) $\bar{Uc}$/molecule as a function of the biaxial strain on graphene with different carrier concentrations of graphene. d) $\bar{Uc}$/molecule as a function of the carrier concentration with different graphene strains. The + and − signs of n_0_ indicate electrons and holes, respectively.

We proposed a mechanism based on our observations that can explain the absence of nucleation and growth of C_60_ thin films at the apex regions. Electron transfer is known to occur between graphene and C_60_ molecules when the Fermi level of graphene (*E*_F_) aligns with or lies higher than the lowest unoccupied molecular orbital (LUMO) level *E*_LUMO_ of C_60_ (**Figure** **5**a).^[^ ^41^, ^42^ ^]^ When an electron is transferred from graphene to C_60_, Coulomb attraction between the negatively charged C_60_ and the positively charged graphene is added to their van der Waals interaction.^[^ ^42^, ^43^ ^]^

One of the consequences of strain‐induced pseudo‐scalar potential is a shift of the Dirac point energy *E*_D_ as

(1) $$E_{D}=E_{D0}\;-g_{\varepsilon }\left(\varepsilon_{xx}+\varepsilon_{yy}\right)$$

where *E*_D0_ is the Dirac point energy of unstrained graphene (−4.5 eV), ε_*xx*_ and ε_*yy*_ are diagonal components of strain tensors, and *g*_ε_ is a constant. The reported theoretical and experimental values of *g*_ε_ are in the range between 2.5 and 4.1 eV;^[^ ^44^ ^]^ *g*_ε_ =  3.8 eV^[^ ^21^ ^]^ is used in this analysis. The *E*_F_ of graphene can be modulated by strain‐induced pseudo‐electric fields; thus, the amount of charge transfer and, eventually, the Coulomb attraction between graphene and C_60_ molecules can be determined by the strain in graphene. If the surface concentration of C_60_ on the graphene is sufficiently low, C_60_ molecules can be treated as a point charge −*e* and graphene can be treated as an infinitely flat conducting surface with a total charge +*e*. The Coulombic energy *U*_C_ per C_60_ molecule can then be estimated using the image‐charge method as

(2) $$U_{C}=\frac{1}{4\pi \varepsilon_{0}}\;\frac{e^{2}}{2d}$$

where ε_0_ is the vacuum permittivity and *d* is the distance between graphene and the center of the C_60_ molecule (Figure 5b); *d* has been reported to be 6.53 Å,^[^ ^6^ ^]^ which gives *U*_C_ = 1.1 eV. The LUMO levels of C_60_ on graphene are not a definite value but have distributions; thus, electron transfer between graphene and C_60_ occurs only for a fraction of the C_60_ molecules even though the average LUMO level of C_60_ molecules ($\bar{E_{\mathrm{LUMO}}}$) lies lower than the *E*_F_ of graphene. If *Pr* is the probability of electron transfer between graphene and C_60_, then the average Coulombic energy $\bar{U_{C}}$ per molecule for many C_60_ molecules on graphene is given by

(3) $$\bar{U_{C}}=\;PrU_{C}$$

To calculate *Pr*, for simplicity, we assumed that the probability distribution of LUMO levels of C_60_ on graphene has a normal distribution $G(E;\bar{E_{\mathrm{LUMO}}},\sigma )$, where *E* is the electronic energy level of C_60_, $\bar{E_{LUMO}}$= −5.0 eV^[^ ^45^ ^]^, and σ = 0.1 eV^[^ ^46^ ^]^ is their standard deviation. Then, *Pr* would be the probability that the LUMO level of a C_60_ molecule is equal to or deeper than *E*_F_(ε_bi_):

(4) $$Pr\left(\varepsilon_{\mathrm{bi}}\right)\;=\int_{-\infty }^{E_{F}\left(\varepsilon_{\mathrm{bi}}\right)}G\left(E;\bar{E_{\mathrm{LUMO}}},\sigma \right)dE$$

where $\varepsilon_{\mathrm{bi}}=\frac{1}{2}\;\left(\varepsilon_{xx}+\varepsilon_{yy}\right)$ is the biaxial strain in graphene. The *Pr* of unstrained graphene as a function of the electron concentration in graphene (*n*_0_) was calculated first (Figure S14, Supporting Information). The results show that *Pr* was <1% when *n*_0_ < −4.38 × 10^12^ cm^−2^. Here, the negative value of *n*_0_ means the graphene is hole‐doped. This critical *n*_0_ value numerically matches a previously reported experimental critical value (− 4.4 × 10^12^ cm^−2^);^[^ ^5^ ^]^ this agreement justifies our choices of $\bar{E_{\mathrm{LUMO}}}$, σ, and *E*_D0_. The *E*_F_ of graphene depends on strain‐induced pseudo‐electric fields in graphene (i.e., *E*_F_ (*n*_0_,ε_bi_) =   − 4.5 − Δ*E*_F_(*n*_0_) − 2*g*_ε_ε_bi_ [eV], where Δ*E*_F_(*n*_0_) is the Fermi level shift due to doping); thus, *Pr* also depends on ε_bi_. Combining Equation (2) with Equation (4), we can calculate $\bar{U_{C}}$ as a function of *n*_0_ and biaxial strain ε_bi_ as

(5) $$\bar{U_{C}}\left(n_{0},\varepsilon_{\mathrm{bi}}\right)\;=\frac{1}{4\pi \varepsilon_{0}}\;\frac{e^{2}}{2D}\int_{-\infty }^{E_{F}\left(n_{0},\varepsilon_{\mathrm{bi}}\right)}G\left(E;\bar{E_{\mathrm{LUMO}}},\sigma \right)dE$$

$\bar{U_{C}}$ as a function of ε_bi_ for various initial doping levels of graphene (Figure 5c) and as a function of *n*_0_ for various ε_bi_ of graphene were calculated using Equation (5) (Figure 5d). The results demonstrated that tensile strain in graphene can reduce the activation energy required for C_60_ desorption from the graphene surface. This corresponds with the inference drawn from the growth behavior of C_60_ on G/NS‐array templates.

### 2.4 Atomistic Processes of C_60_ Thin Film Growth on Strained Graphene Template

**Figure 6:**
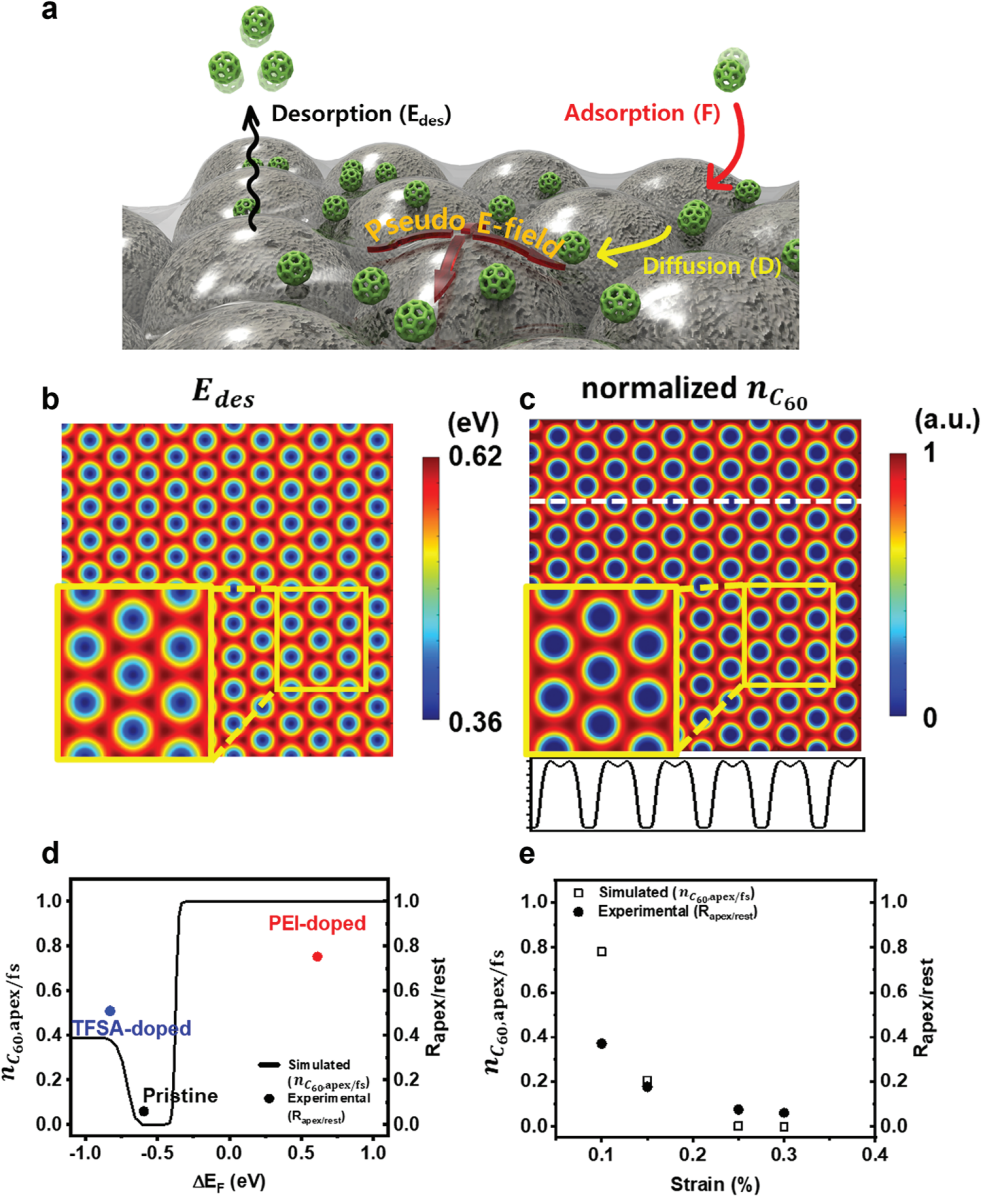
Mechanism of the growth of C_60_ on graphene under strain‐induced pseudo‐electric fields. a) Schematic drawing of the nucleation process involving diffusion, adsorption, and desorption of C_60_ molecules on graphene. b) FDM‐modelled desorption energy profiles on a 2000 nm × 2000 nm G/NS‐array. c) Calculated concentration of C_60_, which was normalized by scaling between 0 to 1. d) Ratio of the normalized concentration of C_60_ at the apex regions to that at the free‐standing regions ($n_{c_{60},\mathrm{apex}/\mathrm{fs}}$) as a function of ΔE_F_. ΔE_F_ is the Fermi level shift with respect to E_D_ due to doping, alongside the *R*_apex/rest_. e) $n_{c_{60},\mathrm{apex}/\mathrm{fs}}$ as a function of the average strain of G/NS‐arrays alongside the *R*_apex/rest_.

Then, we investigated how this reduction in *E*_des_ would affect the surface concentration of C_60_ ad‐molecules ($n_{C_{60}}$) prior to nucleation (**Figure** **6**a). For this purpose, we used FDM to solve the diffusion equation in two dimensions:

(6) $$\frac{\partial n_{C_{60}}\left(x,y,t\right)}{\partial t}=\;-\nabla \cdot \vec{J}-\frac{n_{C_{60}}\left(x,y,t\right)}{\tau_{C_{60}}\left(x,y\right)}+F$$

(7) $$\vec{J}=\;-D\left(x,y\right)\nabla n_{C_{60}}\left(x,y,t\right)$$

where $\vec{J}$ is the diffusion flux of C_60_ molecules, *D* is the diffusivity, $\tau_{C_{60}}\left(x,y\right)\;=10^{-13}\;\exp\left(\frac{E_{\mathrm{des}}\left(x,y\right)}{k_{B}T}\right)$ is the lifetime of C_60_ ad‐molecules where *k*_B_ is the Boltzmann constant and *T* is the absolute temperature, and *F* is the deposition rate. In our simulation, *D*  =  1.5 × 10^−10^ m^2^/s^[^ ^47^ ^]^ and the boundary conditions were $\vec{J}=\;0$ at the boundaries. The positions of C_60_ nuclei on the bare NS‐array were distributed randomly (Figure S8, Supporting Information); thus, *F* was reasonably assumed to be a constant independent of the position. We used *F*  =  1.185 × 10^−8^ mol/(m^2^ · s), which is equivalent to 0.1 Å/s. *E*_des_(*x*,*y*) as a function of position was modeled as a sinusoidal function with the same periodicity as the NS‐array (Figure 4b, details in Supporting Information). For simplicity, the desorption energies at the apex regions (*E*_des,apex_) and free‐standing regions (*E*_des,fs_) were modelled as a weighted sum of the van der Waals interactions (${\bar{U}}_{\mathrm{vdW}}$) and Coulombic interactions (${\bar{U}}_{C}$) between C_60_ and graphene:

(8) $$E_{\mathrm{des},\mathrm{apex}}=\;\alpha {\bar{U}}_{\mathrm{vdW}}\left(\varepsilon_{\mathrm{bi}}\right)+{\bar{U}}_{C}\left(n_{0},\varepsilon_{\mathrm{bi}}\right)$$

(9) $$E_{\mathrm{des},\mathrm{fs}}=\;\alpha {\bar{U}}_{\mathrm{vdW}}\left(\;\varepsilon_{\mathrm{bi}}=\;0\right)+{\bar{U}}_{C}\left(n_{0},\;\varepsilon_{\mathrm{bi}}=\;0\right)$$

The fitting parameter α  =  0.25 was introduced to account for the reduction in ${\bar{U}}_{\mathrm{vdW}}$ due to the out‐of‐plane thermal vibration of the graphene surface.^[^ ^6^, ^48^ ^]^ In this simplified model, ${\bar{U}}_{\mathrm{vdW}}$ is assumed to depend only on ε_bi_ and is unaffected by the curvature (see Figure S12, Supporting Information) or graphene doping level.^[^ ^49^ ^]^ The estimated ${\bar{U}}_{\mathrm{vdW}}\;\left(\varepsilon_{\mathrm{bi}}\right)=\;1.1368-3.155\varepsilon_{bi}$ was derived from the DFT calculation results (Figure S12, Supporting Information) and ${\bar{U}}_{C}\left(n_{0},\varepsilon_{\mathrm{bi}}\right)$ was calculated using Equation (5).

Figure 6b,c shows the calculated 2000 nm × 2000 nm *E*_des_(*x*,*y*) and $n_{C_{60}}\left(x,y,t\right)$ at *t* = 100 ns on the G/NS‐array templates with 200 nm nanospheres. The experimental values of *n*_0_ (−2.5 × 1012 cm^−2^) and ε_*bi*_ at the apex regions (3.1%) were employed in this analysis. As shown in Figure 6b, the calculated *E*_des,apex_ was 0.26 eV lower than *E*_des,fs_. This discrepancy arises because of the presence of tensile strain within graphene in the apex regions. Therefore, C_60_ desorbed more often from the apex regions than from the free‐standing regions. The spatial variation in the desorption energy resulted in a substantial spatial variation in $n_{C_{60}}$ (Figure 6c). C_60_ molecules were sparser in the apex regions than in the free‐standing regions. Specifically, $n_{C_{60}}$ at the apex region was calculated to be 0.03% of that calculated in the free‐standing region. These model simulation results are consistent with our experimental observations where the C_60_ nuclei density was considerably lower in the apex regions than in the freestanding regions.

Additionally, the same diffusion equation was solved to simulate $n_{C_{60}}\left(x,y,t\right)$ at *t* = 100 ns on the same G/NS‐array templates (ε_bi_ at the apex = 3.1% and NS diameter = 200 nm) but with different *n*_0_ values. The ratio of $n_{C_{60}}$ at the apex regions to that at the free‐standing regions ($n_{C_{60},\mathrm{apex}/\mathrm{fs}}$) was calculated as a function of Δ*E_F_* (Figure 6d) to compare the simulation outcomes with the experimental observations. The $n_{C_{60},\mathrm{apex}/\mathrm{fs}}$ ratio was compared with the experimental *R*_apex/rest_ values obtained using the G/TFSA/NS‐ and G/PEI/NS‐array templates. A direct quantitative comparison between the simulated $n_{C_{60},\mathrm{apex}/\mathrm{fs}}$ and experimental *R*_apex/rest_ is difficult because the nucleation and growth of C_60_ are affected by various factors and not solely by $n_{C_{60}}$. Nonetheless, the dependence of the $n_{C_{60},\mathrm{apex}/\mathrm{fs}}$ ratio on the Δ*E_F_* of graphene qualitatively agrees with that of the experimental *R*_apex/rest_. This finding supports the fact that our model effectively captures the fundamental attributes of real‐world scenarios.

When Δ*E_F_* ≈ −0.5 eV, where the *E*_F_ of unstrained graphene is similar to the $\bar{E_{\mathrm{LUMO}}}$ of C_60_, the effect of ε_bi_ on the suppression of C_60_ nucleation at the apex regions is predicted to be prominent ($n_{C_{60},\mathrm{apex}/\mathrm{fs}}\approx 0$). The presence of ε_bi_ specifically at the apex regions of graphene induces a shift in the *E*_F_ of the graphene at the apex regions due to the strain‐induced pseudo‐ electric field. This shift leads to a reduction in the $\bar{U_{C}}$ and, thus, the desorption of C_60_ at the apex regions.

As Δ*E_F_* increased, $n_{C_{60},\mathrm{apex}/\mathrm{fs}}$ saturated at 1. The n‐doping‐induced upshift of the *E_F_* of graphene effectively enabled a nearly complete charge transfer between graphene and C_60_, even when the apex regions of the G/NS‐array templates were subjected to tensile strain. Therefore, the difference in $\bar{U_{C}}$ between the apex and free‐standing regions was negligible. In contrast, $n_{C_{60},\mathrm{apex}/\mathrm{fs}}$ saturated at 0.4 as the graphene became p‐doped. When graphene was significantly p‐doped, charge transfer was suppressed not only at the apex regions, but also in the free‐standing regions. Thus, the $\bar{U_{C}}$ at the apex regions and that at the freestanding regions were inconsequential. Therefore, the calculated $n_{C_{60},\mathrm{apex}/\mathrm{fs}}$ below 1 for considerably p‐doped graphene can be attributed to the influence of ${\bar{U}}_{\mathrm{vdW}}$, which exhibits a weak dependence on the graphene strain level.

Furthermore, we conducted calculations for $n_{C_{60}}\left(x,y,t\right)$ at *t* = 100 ns on G/NS‐array templates, employing varying NS diameters ranging from 50 to 500 nm. The value of *n*_0_ was consistently set to −2.5 × 1012 cm^−2^, and the corresponding experimental ε_bi_ values at the apex regions were used for the distinct G/NS‐array templates (and Figure S15, Supporting Information). The dependence of $n_{C_{60},\mathrm{apex}/\mathrm{fs}}$ on ε_bi_ at the apex regions was qualitatively similar to that of the experimentally derived *R*_apex/rest_ (Figure 6e).

We also solved the same diffusion equation but with position‐dependent *D*(*x*, *y*) and position‐independent $\tau_{C_{60}}$. In this case, the obtained $n_{C_{60}}\left(x,y,t\right)$ was spatially uniform (Figure S16, Supporting Information). Therefore, we concluded that the position‐dependent diffusivity of C_60_, which might be caused by the strain field in graphene, cannot attribute to preferential nucleation of C_60_ on the G/NS‐array.

The simulation results shown in Figures 5 and 6 the observed nucleation behavior of C_60_ on graphene/NS‐array templates may be a consequence of the reduction in the *E*_des_ of C_60_, which was caused by the strain‐induced pseudo‐electric fields in graphene at the top of the NSs.

## 3 Conclusion

We investigated how strain fields in a graphene template affect the nucleation and growth of a C_60_ thin film. When graphene was transferred onto a silica NS array, tensile strain was developed in the graphene directly in contact with the tops of the NSs; the other part of the graphene was free‐standing between them. C_60_ ad‐molecules rapidly desorbed from the apex regions of the G/NS‐array templates; thus, the C_60_ thin film nucleated preferentially on the free‐standing regions. Based on our observations, we proposed a growth mechanism of C_60_ on G/NS‐array templates. Tensile strain in the apex regions of graphene leads to the generation of pseudo‐electric fields that shift the Fermi energy (E_F_) of graphene to a level below the Lowest Unoccupied Molecular Orbital (LUMO) level of C_60_, thereby preventing the transfer of electrons from the graphene to the C_60_ ad‐molecules. The absence of electron transfer led to a decrease in Coulombic attraction between the graphene and C_60_ molecules, and this change was sufficient to promote the desorption of C_60_ molecules from the graphene.

Our results provide insights into the strain engineering of graphene templates to enable the growth of OSC thin films with the desired structure and properties. When combined with strain‐engineering of other 2D materials such as hexagonal boron nitride and 2D transition‐metal dichalcogenides,^[^ ^50^, ^51^, ^52^, ^53^ ^]^ our findings might lead to the development of next‐generation optoelectronic devices that use strain‐engineered organic/2D material hybrid materials.

## Conflict of Interest

The authors declare no conflict of interest.

## Supporting information

Supporting Information

## Data Availability

Research data are not shared.
