# Supplementary material for: Self‐Assembly of Organic Semiconductors on Strained Graphene under Strain‐Induced Pseudo‐Electric Fields
Source: Adv Sci (Weinh). 2024 Mar 13;11(19):2400598. doi: 10.1002/advs.202400598 (PMC11109627; doi:10.1002/advs.202400598)
Supplement: Supplementary file 1 — Supporting Information [file ADVS-11-2400598-s001.pdf]

ADVANCED SCIENCE
Open Access

# Supporting Information

for *Adv. Sci.*, DOI 10.1002/advs.202400598

## Self-Assembly of Organic Semiconductors on Strained Graphene under Strain-Induced Pseudo-Electric Fields

*Jinhyun Hwang, Jisang Park, Jinhyeok Choi, Taeksang Lee, Hyo Chan Lee* and Kilwon Cho**

Supporting Information

**Self-Assembly of Organic Semiconductors on Strained Graphene under Strain-Induced Pseudo-Electric Fields**

*Jinhyun Hwang, Jisang Park, Jinhyeok Choi, Taeksang Lee, Hyo Chan Lee* and Kilwon Cho**

## Experimental methods

*Fabrication of silica nanosphere array.*

200 nm silica nanospheres with 10mg/mL dispersed in DI water were purchased from nanoComposix (SISN200-25M). 300nm thick $SiO_2$/Si substrates was cleaned by isopropanol, acetone, and UVO treatment. Spin-coating of silica nanospheres on $SiO_2$/Si substrates was performed in three steps to enhance size of NS cluster and degree of close-packing of NS-array.[1] Nanospheres were coated by optimal condition 200-500-1500 rpm and 30-30-60 s respectively.

*Synthesis and transfer of graphene.*

Graphene was synthesized on a copper foil by chemical vapor deposition as described elsewhere.[2] PMMA in chlorobenzene was spin-coated for 3000rpm 60sec and baked for 30min 120 °C to remove residual solvent. $O_2$ plasma ion removes back-sided graphene on a copper foil using a reactive ion etcher. Diluted ammonium persulfate solvent (0.05M) etched copper foil and rinsed by floating on DI water for 1 hour. UVO treatment was applied NS-array on 300nm $SiO_2$/Si wafer for 30 min. Graphene was carefully transferred on NS-array and dried in moderate temperature vacuum condition to prevent graphene rupturing on NS-array. Transferred graphene was immersed into acetone for 3 hours and hydrogen was annealed at 400 °C for 2 hours to remove PMMA residue.

*Bottom-side doping method*

The transfer process from Cu etching to floating in DI water was identical to that of the previous transfer method. Subsequently, the graphene was placed on a water-based solution containing either p-type bis(trifluoromethanesulfonyl)amide (TFSA) or n-type poly(ethylene imine) (PEI) dopants for 180 s. The TFSA solution was prepared by blending 5 mL of $20 \times 10^{-3}$ M TFSA in nitromethane and 50 mL of DI water, whereas the PEI solution was created

by mixing 1 mL of 5 × $10^{-3}$ M PEI in ethanol and 50 mL of DI water.[3] Finally, the doped graphene film was transferred to an NS array.

*$C_{60}$ deposition.*

$C_{60}$ was deposited using the same methodology as previously employed.[4] Organic molecular beam deposition (OMBD) was used to deposit $C_{60}$ (Aldrich Chemicals, 99.99% purity) in ultra-high vacuum (UHV, $10^{-8}$ Torr). The substrate temperature was kept at room temperature, and the deposition rate was 0.1Å/s.

**Calculation of Diffusion Equation**

The following diffusion equation is calculated by Finite-Difference-Method using MATLAB:

$$\frac{\partial n_{\mathrm{C}_{60}}(x,y,t)}{\partial t} = -\nabla \cdot \vec{J} - \frac{n_{\mathrm{C}_{60}}(x,y,t)}{\tau_{\mathrm{C}_{60}}(x,y)} + F, \quad \text{(Eq. S1)}$$

$$\vec{J} = -D(x,y)\nabla n_{\mathrm{C}_{60}}(x,y,t), \quad \text{(Eq. S2)}$$

where $n_{C_{60}}$ is surface concentration of $C_{60}$ ad-molecules, $\vec{J}$ is the diffusion flux of $C_{60}$ molecules, $D(x,y)$ is diffusivity, $\tau_{\mathrm{C}_{60}}(x,y)$ is the lifetime of $C_{60}$ ad-molecules, and $F$ is the deposition rate. $F$ was assumed to be constant, $F = 1.185 \times 10^{-8}\ mol/m^2 \cdot s$ which is equivalent to 0.1Å/s.

For calculation of $n_{\mathrm{C}_{60}}(x,y,t)$ when $\tau_{\mathrm{C}_{60}}(x,y)$ is a function of position, we first modeled position-dependent desorption barrier:

$$E_{\mathrm{des}}(x,y) = \frac{1}{2}\ (E_{\mathrm{des,0}} + E_{\mathrm{des,apex}}) - \frac{1}{2}\left(E_{\mathrm{des,0}} - E_{\mathrm{des,apex}}\right)\left\{1 - \frac{4}{9}(3 - \textstyle\sum_{i=1}^{3} \cos(\overrightarrow{\boldsymbol{b_i}} \cdot \vec{\boldsymbol{r}})\right\},$$
(Eq. S3)

where $E_{\mathrm{des,0}}$ and $E_{\mathrm{des,apex}}$ are desorption barriers of unstrained graphene and that of strained graphene at the apex of NS, respectively, $\overrightarrow{\boldsymbol{b_1}} = \left(\frac{2\pi}{a}, -\frac{2\pi}{\sqrt{3}a}\right), \overrightarrow{\boldsymbol{b_2}} = \left(0, \frac{4\pi}{\sqrt{3}a}\right), \overrightarrow{\boldsymbol{b_3}} = -\overrightarrow{\boldsymbol{b_1}} - \overrightarrow{\boldsymbol{b_2}}$, and $\vec{\boldsymbol{r}} = (x,y)$. Then, the lifetime is calculated by the equation $\tau_{\mathrm{C}_{60}}(x,y) = 10^{-13}\exp\left(\frac{E_{\mathrm{des}}(x,y)}{k_B T}\right)$ where $k_B$ is Boltzmann constant and $T$ is temperature. In our simulation, $D = 1.5 \times 10^{-10} m^2/s$, and the boundary conditions were $\vec{J} = 0$ at the boundaries.

For calculation of $n_{\mathrm{C}_{60}}(x,y,t)$ when diffusivity is a function of position (**Figure S16**), we modeled position-dependent diffusivity as follow:

$$D(x,y) = D_{\mathrm{avg}} + D_{\mathrm{amp}}\left\{1 - \frac{4}{9}(3 - \textstyle\sum_{i=1}^{3} \cos(\overrightarrow{\boldsymbol{b_i}} \cdot \vec{\boldsymbol{r}})\right\} \quad \text{(Eq. S4)}$$

where $D_{avg}$ is the average diffusion coefficient over the whole space and $D_{amp}$ is the amplitude.

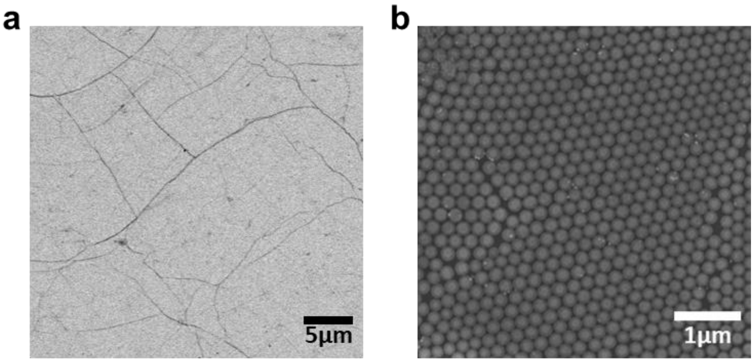


**Figure S1.** SEM images of a) graphene/flat $SiO_2$ (G/Flat $SiO_2$) and b) graphene/NS-array (G/NS-array).

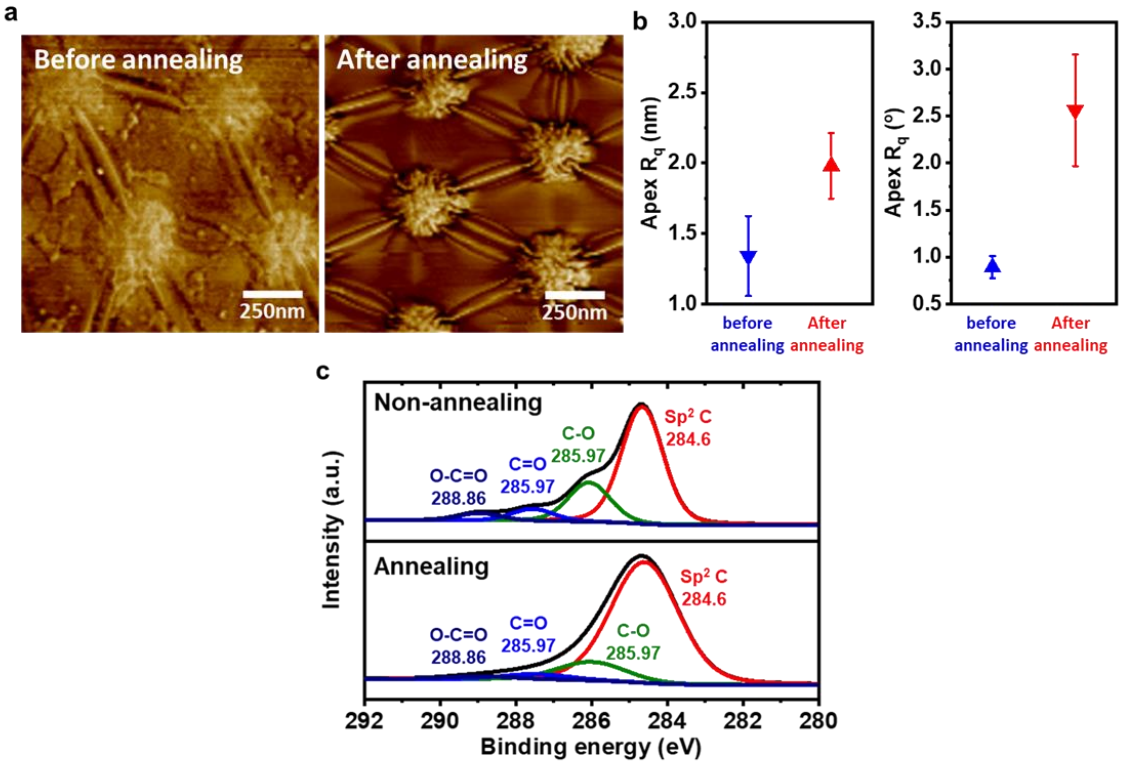


**Figure S2.** a) AFM phase images of G/NS-array before and after annealing in hydrogen atmosphere. b) RMS roughnesses $R_q$ of height (left) and phase (right), and c) XPS C1s peaks before and after annealing in hydrogen atmosphere.

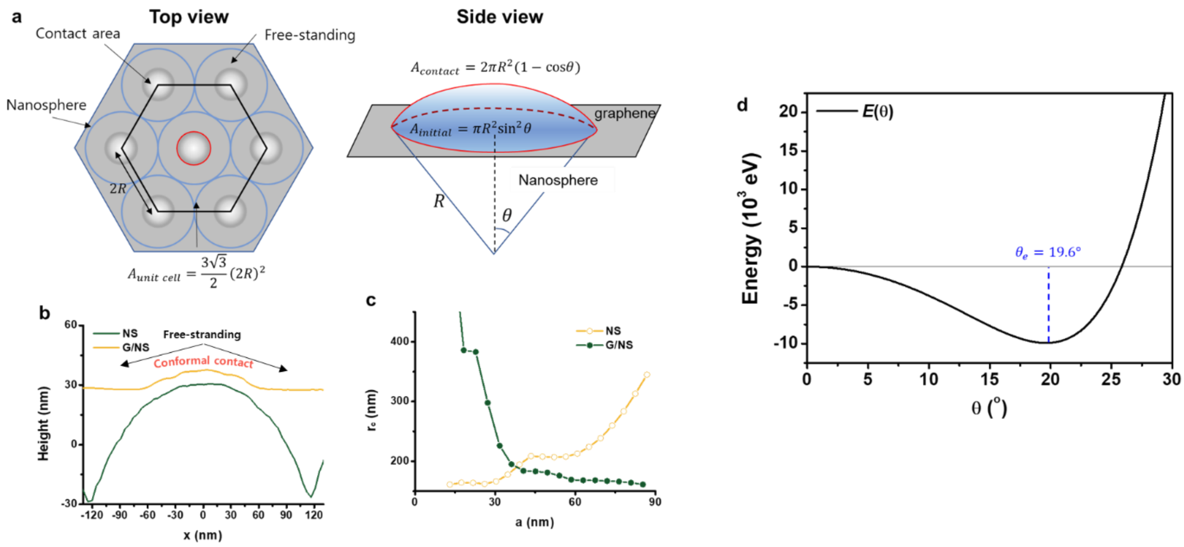


**Figure S3.** Schematic diagram and AFM data of topographic information on G/NS-array. a) Top and side view unit-cell of G/NS-array y. b) Overlap of cross-section image obtained from AFM height profile. c) The average radius of curvature of NS (white) and G/NS-array (green). d) Total energy of G/NS-array system per one nanosphere as a function of $\theta$.

The radius $R$ of NS was estimated to be ~ 121 nm (**Figure S3**a, b). Then, each average radius of curvature ($r_c$) of bare NS and graphene/NS were estimated by fitting the height profiles shown in Figure S3a with the equation $y = y_0 + \sqrt{r_c{}^2 - x^2}$ in the range of $[-a, a]$ (Figure S3b). Because of blunt tips, the curvature of NS and G/NS-array obtained from AFM might be different from the real cases, especially when $a$ is small. As $a$ becomes larger than 39 nm, $r_c$ of G/NS-array surface started to be larger than the radius of curvature of the bare NS surface (Figure S3c). From this information, we concluded that graphene had conformal contact with NS when $a \leq$ 39 nm. Compared with Figure S3a, $a$ = 39 nm corresponds to $R\sin\theta$. The areal fraction of graphene conformally contact with NS ($f_C$) is equal to the ratio of $3 \times A_{\text{initial}}$ to the area of a hexagonal unit cell ($A_{unit\ cell}$) shown in Figure S3a, that is, $f_C = (3 \times A_{\text{initial}})/A_{\text{unit cell}}$). The areal fraction of graphene conformally contact with NS is 0.096 and corresponding polar angle $\theta$ is 18.8°.

The area of deformed graphene on a NS is $A_{\text{contact}} = 2\pi R^2(1 - \cos\theta)$ and the initial area of the same region before the deformation is $A_{\text{initial}} = \pi R^2 \sin^2\theta$. Here, we assumed that initial graphene before deformation is perfectly flat. This is a highly plausible assumption because very stiff and thus flat PMMA/graphene layer first covers the NS-array, then the deformation of graphene begins with the removal of PMMA layer. So, the applied biaxial strain in graphene can be calculated using the equation,

$\varepsilon = \frac{2\pi R^2(1-\cos\theta)}{\pi R^2 \sin^2\theta} - 1$, (Eq. S5)

The strain in graphene is determined by the competition between the strain energy from the deformation of graphene and the adhesion energy of graphene contact with NS; The increase in energy due to the tensile strain in graphene is compensated by the decrease in energy due to the adhesion energy between graphene and $SiO_2$ surface.[5] The sum of strain energy and van der Waals interaction energy of graphene per one nanosphere is approximately given as,

$E(\theta) \approx \frac{1}{2} E^{2D} \varepsilon^2 \{\pi R^2 sin^2\theta\} - \widetilde{U}_{\mathrm{vdW}}\{2\pi R^2(1 - cos\theta)\}$ (Eq. S6)

where $E^{2D} = 340\ N/m$ is Young's modulus of graphene,[6] and $\widetilde{U}_{\mathrm{vdW}}$the interaction energy between graphene and $SiO_2$, which is reported to be 0.45 J/m$^2$.[7] At equilibrium, $\theta = \theta_e$ is the polar angle that minimizes $E(\theta)$.

Figure S3d shows $E(\theta)$ as a function of $\theta$. As a result, the minimum is at $\theta_e$= 19.6°. At the equilibrium, the strain at the apex region is calculated to be 2.98% according to Eq. S5, and the calculated $f_C$ is 0.102. Lastly, the average biaxial strain of whole area of graphene, which corresponds to the strain in graphene measured by Raman spectroscopy, is calculated to be $f_C\varepsilon$ = 0.30%. The calculated $\theta_e$, $f_C$ and average strain are all numerically consistent with the experimental values (18.8°, 0.096, 0.30%, respectively). Though the model to describe the strain in graphene on NS-array is oversimplified, the numerical agreements of the simple analytical model with the experimental values strongly implies that biaxial strain around 3% is indeed present at the apex region of G/NS-array.

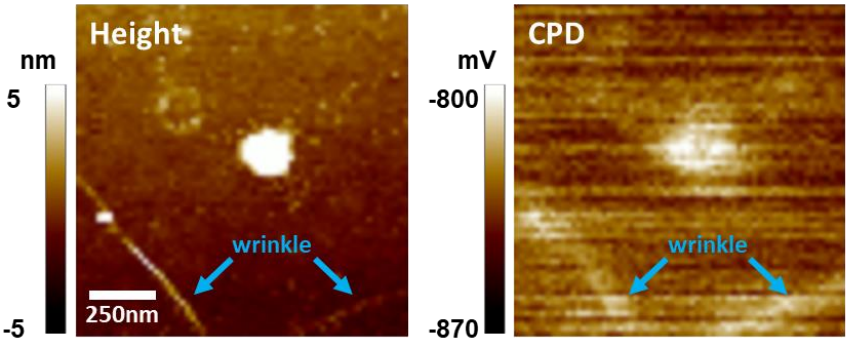


**Figure S4.** Height image (left) and contact potential difference (CPD) image (right) of G/Flat $SiO_2$.

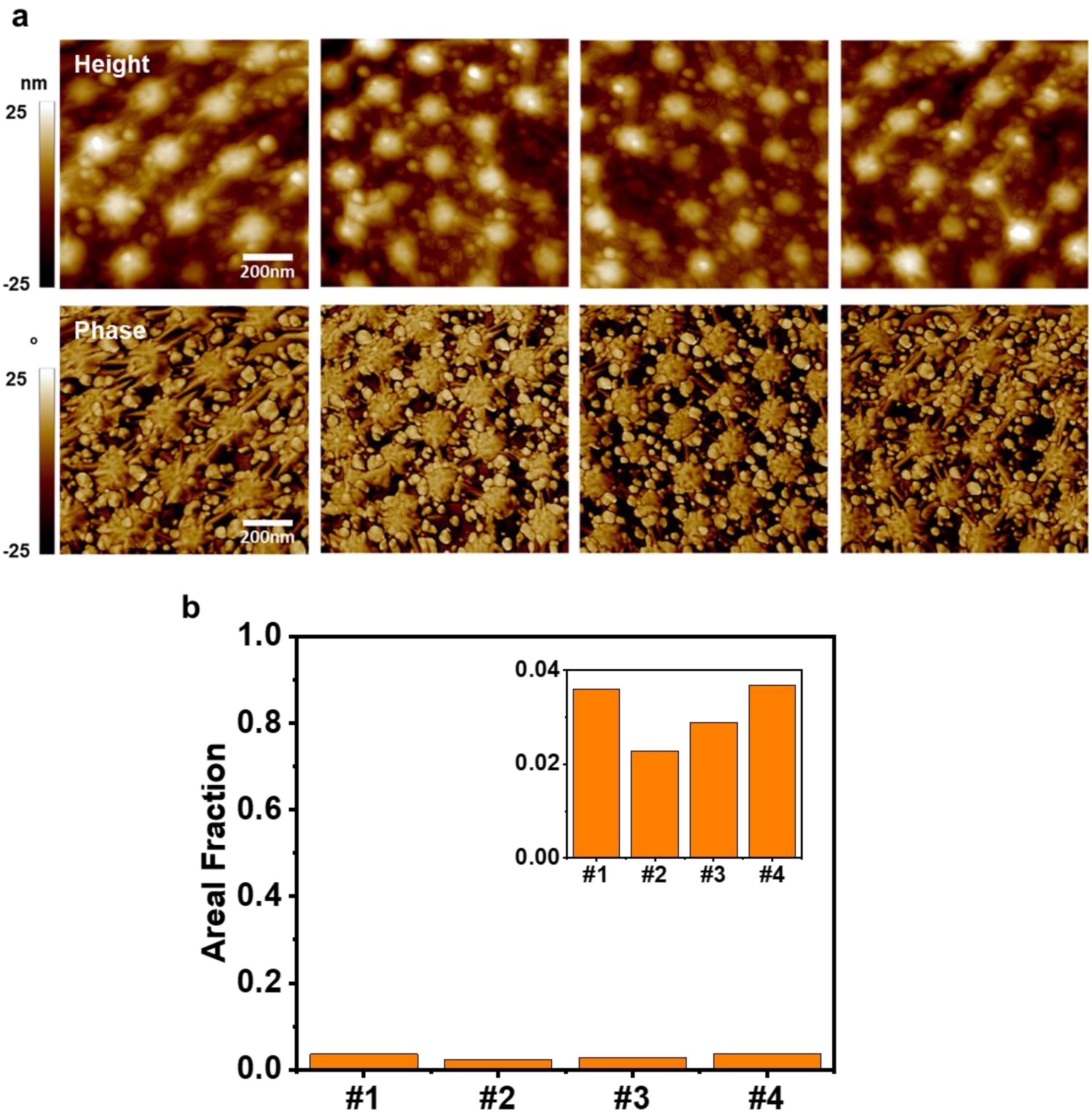


**Figure S5.** a) AFM height images (upper) and phase images (lower) of G/NS-array samples after $C_{60}$ deposition for 40s. b) The fraction of the area of $C_{60}$ islands at the apex regions in G/NS-array templates to the total area of $C_{60}$ islands in G/NS-array samples.

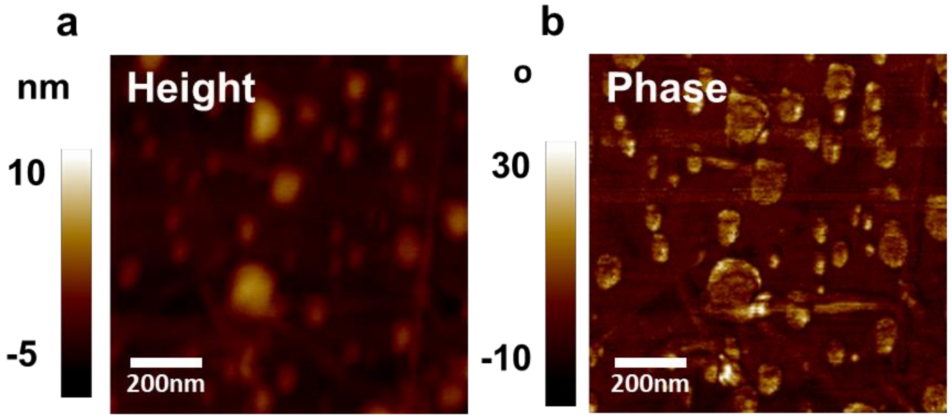


**Figure S6**. AFM a) height and b) phase image of a 0.39 ML $C_{60}$ thin film deposited on G/Flat $SiO_2$.

The nucleation of $C_{60}$ thin films on G/Flat $SiO_2$ was investigated to determine the actual $C_{60}$ deposition rate. $C_{60}$ thin films were deposited on G/Flat $SiO_2$ for 20 s; the deposition rate monitored using QCM was 0.1 Å/s, which is identical to that of the $C_{60}$ on G/NS-array templates in the experiments. The actual volume was determined by adding the volumes of the individual $C_{60}$ islands observed in the AFM images. The total volume was then converted into the nominal thickness of the $C_{60}$ thin films. Consequently, the actual nominal thickness was estimated to be 0.39 ML, which corresponds to a deposition rate of nearly 0.1 Å/s.

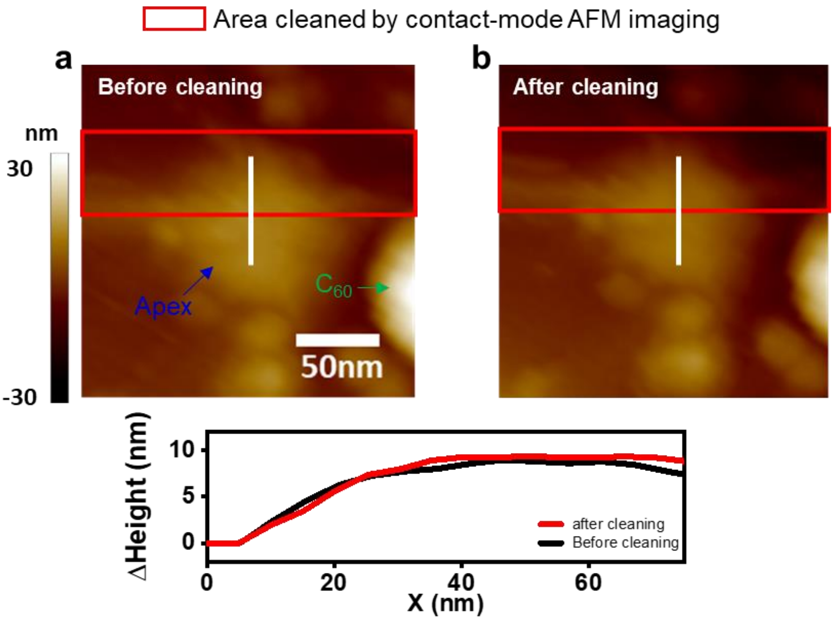

**Figure S7.** AFM height images of an apex region of G/NS-array a) before and b) after contact-mode AFM process. Overlap cross-section before and after AFM scanning shows no difference of height profile.

To verify our observation that $C_{60}$ nucleation was nearly absent at the apex regions of the G/NS-array, we used contact-mode AFM to scan the upper half of an apex region.[8] Normal force of 5nN was applied to prevent any damage to the graphene with conventional tip cantilever (CDT-CONTR, force constant 0.5 N/m). If a $C_{60}$ thin film was present, a corresponding step height would be observed in the height image of the apex region after the scanning process. Figure S7 shows height images of graphene at the apex region before and after the scanning; no step height was observed.

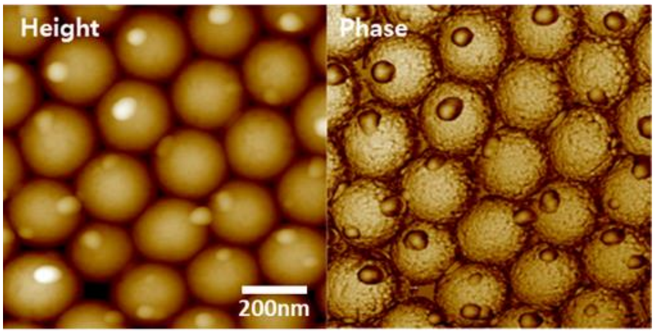


**Figure S8.** Nucleation of $C_{60}$ thin films on bare NS-array.

The observation that $C_{60}$ readily nucleates at the apexes of bare NS-array also excludes the effects of wetting transparency and surface morphology of graphene on the self-assembly of $C_{60}$ molecules. Therefore, we conclude that the strain-induced pseudo-electric fields are responsible for the specific nucleation and growth of $C_{60}$ on the free-standing regions.

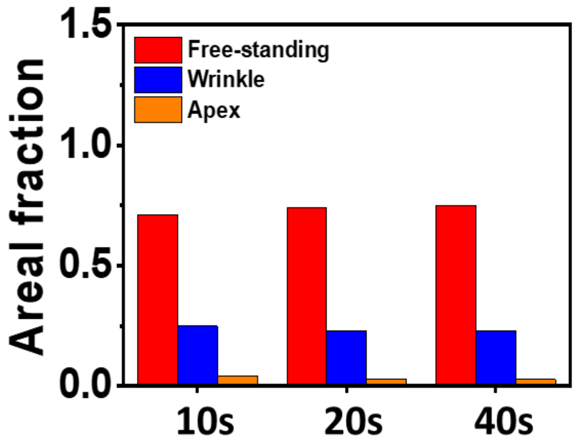


**Figure S9.** Areal fraction of $C_{60}$ islands at the apex regions in G/NS-array templates with a NS diameter of 200nm to the total area of $C_{60}$ islands at the apex (orange), wrinkles (blue), and free-standing (red) regions in the G/NS-array as a function of the deposition time.

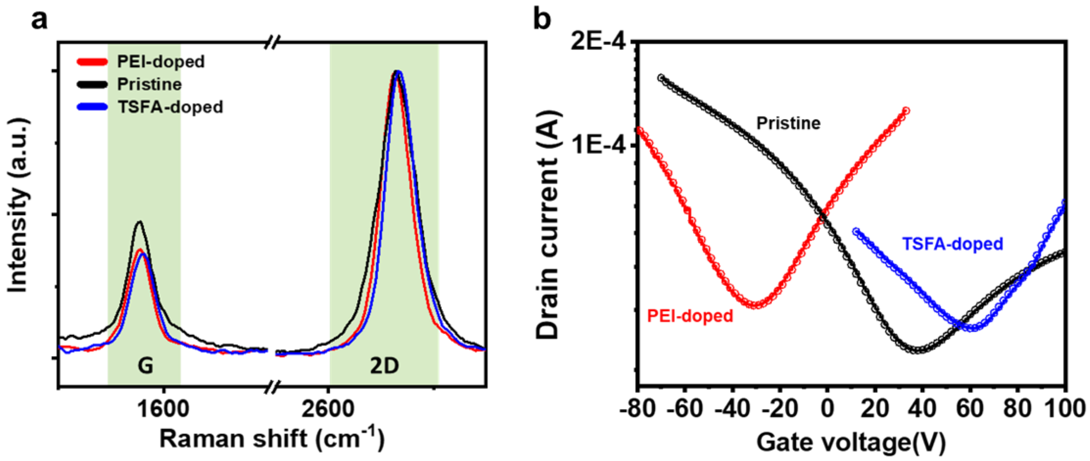


**Figure S10.** a) Raman single spectrum of G/Flat $SiO_2$ with various doping levels. Red line (PEI) and blue line (TFSA) samples were doped using the underside doping method. b) Transfer characteristics of field-effect transistors fabricated with doped graphene. The transfer curves of TFSA- and PEI-contact samples were blue- and red-shifted, respectively, as compared to that of the pristine graphene device. The $SiO_2$ thickness was 300 nm and the drain-source voltage was 0.01 V. The channel length and width were 40 and 1100 μm, respectively.

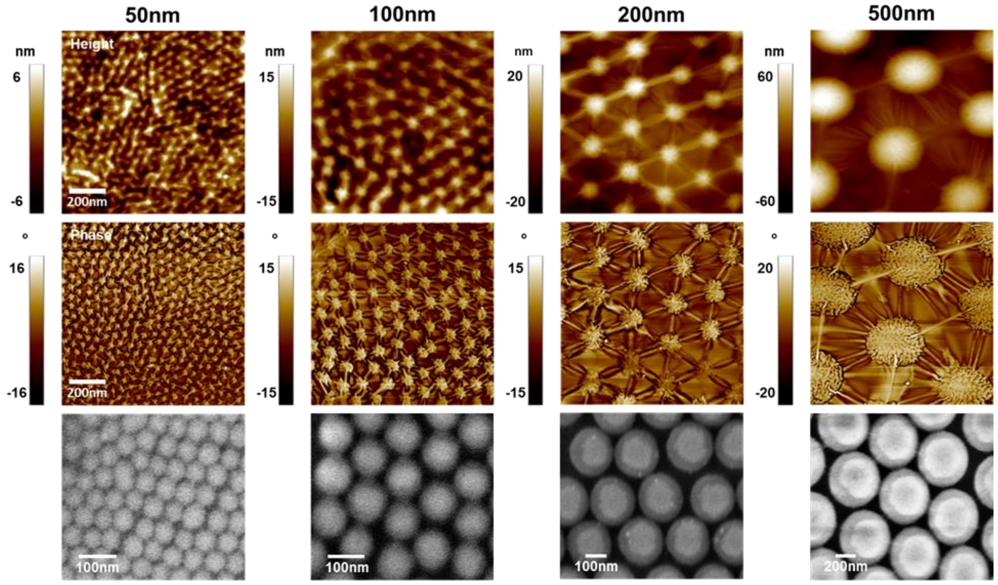


**Figure S11.** AFM height (top row) and phase (middle row) images and SEM images (bottom row) of G/NS-arrays with different nanosphere diameters (50–500 nm).

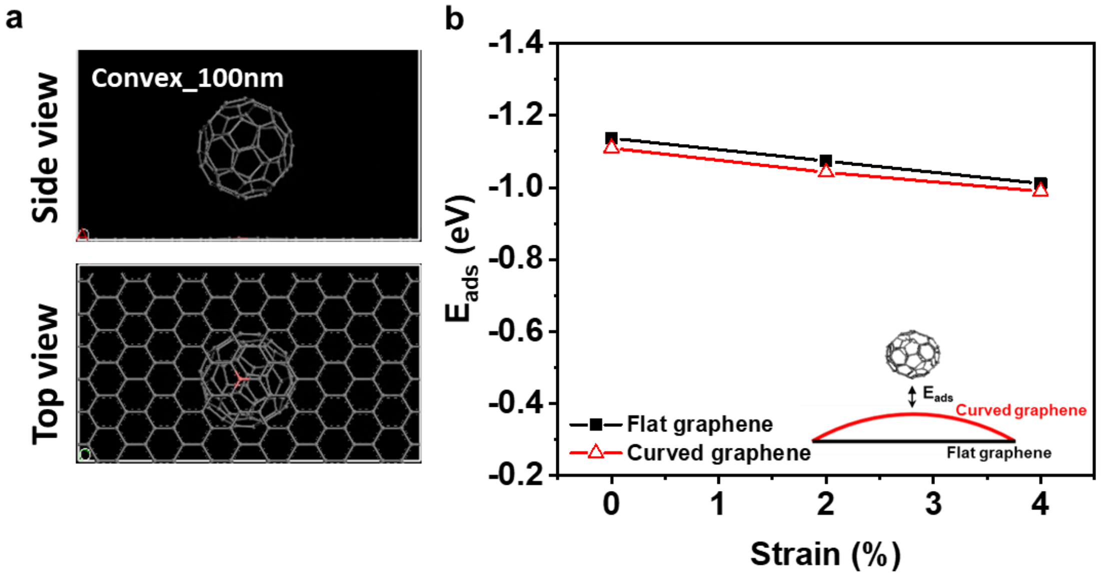


**Figure S12.** DFT simulation of the $C_{60}$ adsorption energy on a graphene surface. a) Side and top views of the $C_{60}$/graphene unit-cell used for the DFT simulation. b) Adsorption energy of $C_{60}$ ($E_{ads}$) as a function of the strain on perfectly flat graphene (black square) and curved graphene (red triangle). The radius of curvature was 100 nm.

DFT calculations were performed at the generalized gradient approximation level in the Perdew−Burke−Ernzerhof (PBE) exchange–correlation functional as implemented in CASTEP in Materials Studio 2020. Dispersion correction to the PBE functional was done within the method of Tkatchenko and Scheffler (DFT-TS) to consider the van der Waals interactions. The ultrasoft pseudo potentials were employed, and all geometry structures were constructed with

20 Å-thick vacuum slabs and fully relaxed using geometric optimization. An energy cutoff of 440 eV and 1 × 1 × 1 k-point meshes for the slabs were used to achieve an energy of $1 \times 10^{-5}$ eV/atom, a force of 0.03 eV/Å, and a displacement convergence of 0.001 Å. The adsorption energies were calculated using the follow equation:

$E_{ads} = E(graphene + fullerene) - E(graphene) - E(fullerene)$ (Eq. S7)

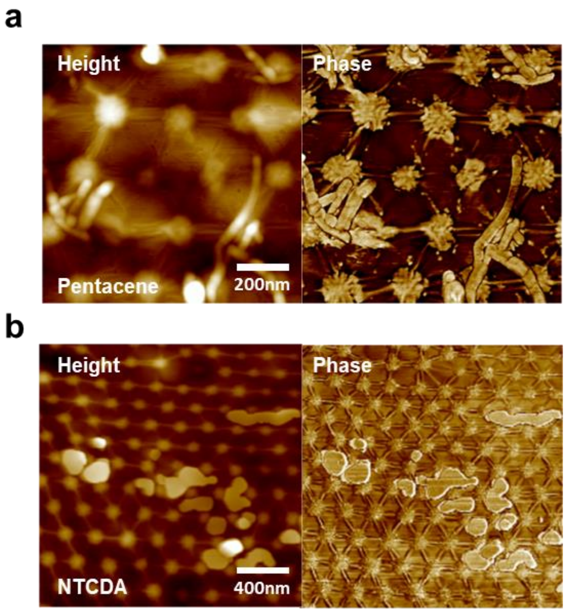


**Figure S13.** Early-stage growth behaviors of a) pentacene and b) 1,4,5,8-naphthalenetetracarboxylic dianhydride (NTCDA) thin films on a G/NS-array with a NS diameter of 200nm.

In contrast to the $C_{60}$/graphene system, the pentacene/graphene system does not exhibit charge transfer upon adsorption because the lowest unoccupied molecular orbital (LUMO) level of pentacene is considerably higher than the $E_F$ of graphene by 1.2–1.3 eV.[9] Because of the large energy gap between the LUMO level of pentacene and the $E_F$ of graphene, charge transfer between pentacene and graphene is expected to be absent, even when graphene is subjected to a moderate tensile strain (a few %).

As shown in Figure S13a, no preferential nucleation or growth of $C_{60}$ was observed in the free-standing regions of the G/NS-array. Therefore, $R_{\mathrm{apex/rest}}$ was estimated to be 1.47, indicating the absence of discernible nucleation or growth of $C_{60}$ in the apex regions, as compared with the free-standing regions. Therefore, the binding energy of pentacene on a graphene/NS-array should be independent of the strain field in the graphene.[10]

Charge transfer between graphene and NTCDA molecules, whose LUMO level is similar to that of $C_{60}$, is expected upon the adsorption of NTCDA molecules. In contrast to pentacene, preferential nucleation and growth were observed in the freestanding regions. $R_{\mathrm{apex/rest}}$ was 0.09.

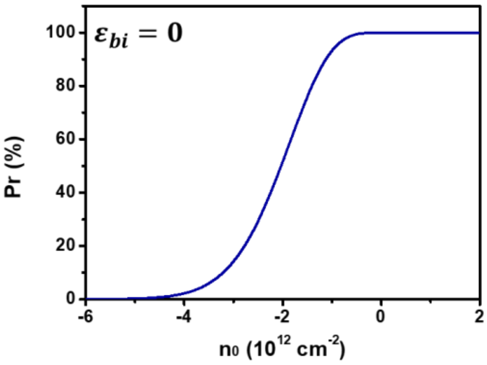


**Figure S14.** Probability of electron transfer from unstrained graphene to a $C_{60}$ ad-molecule.

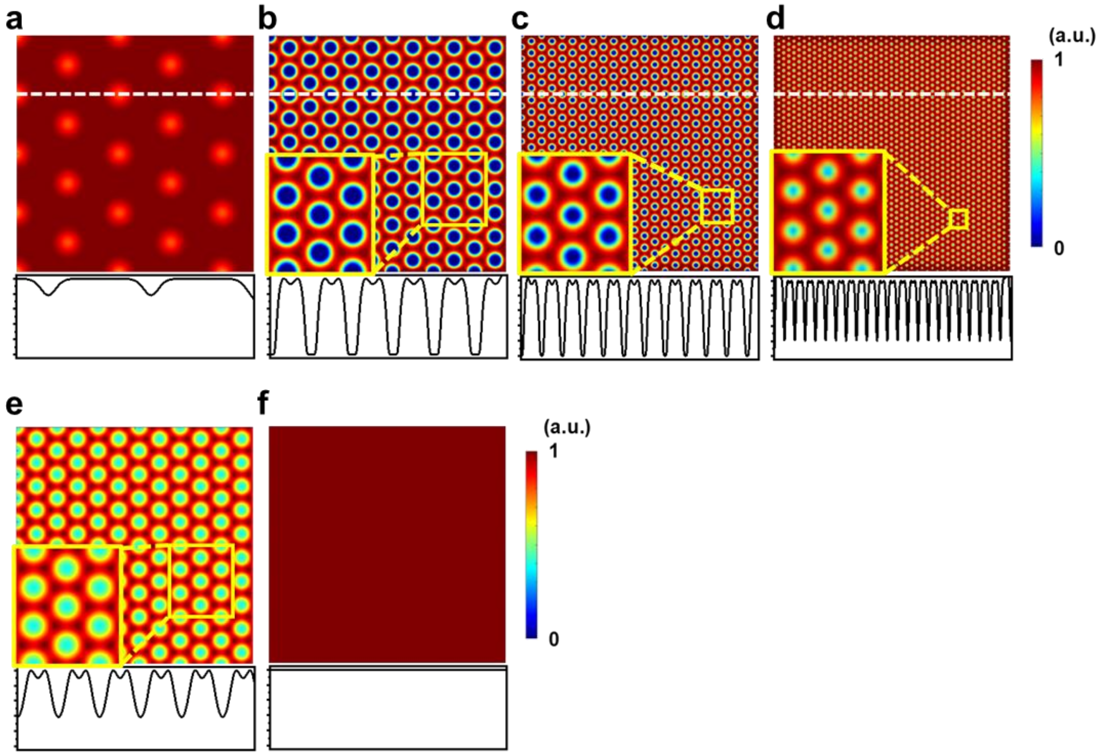


**Figure S15**. Normalized $n_{C60}$ on 2000 nm × 2000nm G/NS-arrays with diameters of a) 500, b) 200, c) 100, and d) 50 nm. Normalized $n_{C60}$ on e) TFSA- and f) PEI-doped G/NS-arrays.

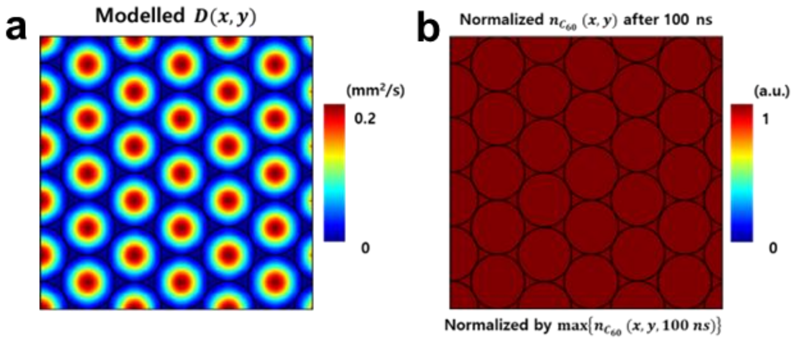


**Figure S16.** a) Finite difference method (FDM)-modeled diffusivity of $C_{60}$ on a G/NS-array with a diameter of 200 nm and b) calculated $n_{C60}(x,y)$ after 100 ns.

| | Strain (%) | Radius of curvature of graphene (nm)* | | | |
|---|---|---|---|---|---|
| | | ∞ | + 100 | + 37.8 | - 86 |
| Binding energy (eV) | 0 | -1.137 | -1.1095 | -1.0736 | -1.1664 |
| | 2 | -1.0733 | -1.0421 | -1.0042 | -1.1095 |
| | 4 | -1.0108 | -0.9896 | -0.9538 | -1.0351 |

**Table S1.** DFT calculation of $E_{ads}$ (eV) as a function of the strain (%) and radius of curvature of graphene. The radius of curvature of 100 nm corresponds to that of 200 nm-diameter nanospheres. The radius of curvature of 37.8 and -86 nm correspond to curvatures of 0.026 and 0.01 $nm^{-1}$, respectively. **(***: + and – signs indicate convex and concave, respectively).

**Supplementary Discussion 1**

**Additional effects of charge transfer between $C_{60}$ and graphene on the nucleation behaviors of $C_{60}$ thin films.**

The charge transfer between $C_{60}$ and graphene causes strong molecule-substrate attraction, which means the reduction of interfacial free energy of $C_{60}$ thin film/graphene. It leads to

smaller energetic barrier $\Delta G^*$ for $C_{60}$ nucleation.[11] In this case, more $C_{60}$ nuclei can be formed on the free-standing regions of G/NS-array where the charge transfer between graphene and $C_{60}$ is expected to occur.

When the charge transfer occurs and thus the interaction between $C_{60}$ molecules and graphene becomes strong, the diffusion barrier of $C_{60}$ molecules on the graphene increases.[12] The limited diffusion of $C_{60}$ ad-molecules can lead to the increase in nucleation density.[13]

The above two arguments cannot explain the absence of $C_{60}$ nucleation on the apex regions of the G/NS-array. However, in addition to the desorption of $C_{60}$ molecules on the apex regions of G/NS-array, they can also be ascribed to the preferential nucleation of $C_{60}$ in the free-standing regions.

**Supplementary Discussion 2**

**Estimation of Coulomb Interaction Contribution to Desorption Energy**

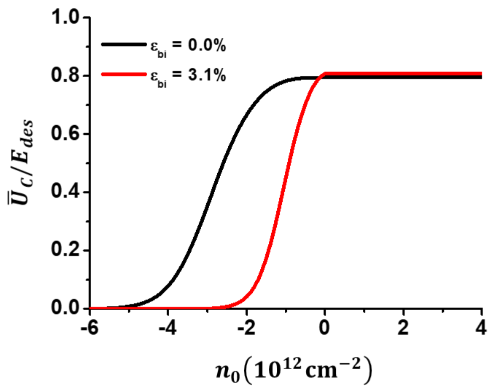


**Figure S17.** $\bar{U}_\mathrm{C}/E_\mathrm{des}$ as a function of $n_0$ when $\varepsilon_\mathrm{bi}$ is 0.0% (black) and 3.1% (red).

In our FDM model, the desorption energy ($E_\mathrm{des}$) was modelled as a weighted sum of the van der Waals interaction ($\bar{U}_\mathrm{vdW}$) and Coulomb interaction ($\bar{U}_\mathrm{C}$) between $C_{60}$ and graphene:

$E_{des} = \alpha \bar{U}_{vdW}(\varepsilon_{bi}) + \bar{U}_{C}(n_0, \varepsilon_{bi}).$ (Eq. S8)

The fitting parameter $\alpha = 0.25$ was introduced to account for the reduction in $\bar{U}_{vdW}$ due to the out-of-plane thermal vibration of the graphene surface. In this simplified model, $\bar{U}_{vdW}$ was assumed to depend only on $\varepsilon_{bi}$. The estimation of $\bar{U}_{vdW}(\varepsilon_{bi}) = 1.1368 - 3.155\varepsilon_{bi}$ was derived from our DFT calculation results (Figure S12) and $\bar{U}_{C}(n_0, \varepsilon_{bi})$ was calculated using Eq. 5 in the main manuscript.

The effect of the Coulombic interaction on the desorption energy (or binding energy) of $C_{60}$ on graphene ($\bar{U}_{C}/E_{des}$, Figure S17) was estimated using Eq. S8. This calculation was performed under conditions in which the graphene was subjected to tensile strains of 0 and 3.1%. Consequently, the Coulombic interaction at a hole concentration of $2.5 \times 10^{12}$ cm$^{-2}$ in graphene ($n_0$=-2.5 × $10^{12}$ cm$^{-2}$) contributed 53 and 0.79% to the desorption energy for the 0 and 3.1% tensile strain conditions, respectively. It is important to note that the quantitative accuracy of our model is not guaranteed as it does not adhere to first-principles theory.

**References**

[1] S. S. Shinde, S. Park, J. Shin, *J. Semicond* **2015**, 36, 043002.

[2] N. N. Nguyen, S. B. Jo, S. K. Lee, D. H. Sin, B. Kang, H. H. Kim, H. Lee, K. Cho, *Nano Lett.* **2015**, 15, 2474.

[3] J. S. Kim, B. J. Kim, Y. J. Choi, M. H. Lee, M. S. Kang, J. H. Cho, *Adv. Mater.* **2016**, 28, 4803.

[4] N. N. Nguyen, H. C. Lee, K. Baek, M. S. Yoo, H. Lee, H. Lim, S. Choi, C.-J. Kim, S. Nam, K. Cho, *Adv. Funct. Mater.* **2021**, 31, 2008813.

[5] W. Gao, P. Xiao, G. Henkelman, K. M. Liechti, R. Huang, *J. Phys. D: Appl. Phys* **2014**, 47, 255301.

[6] C. Lee, X. Wei, J. W. Kysar, J. Hone, *Science* **2008**, 321, 385.

[7] S. P. Koenig, N. G. Boddeti, M. L. Dunn, J. S. Bunch, *Nat. Nanotechnol.* **2011**, 6, 543.

[8] J. Liang, K. Xu, B. Toncini, B. Bersch, B. Jariwala, Y.-C. Lin, J. Robinson, S. K. Fullerton-Shirey, *Adv. Mater. Interfaces* **2019**, 6, 1801321.

[9] Y. Pan, J. Huang, Z. Wang, D. Yu, B. Yang, Y. Ma, *RSC Adv.* **2017**, 7, 26697.

[10] F. Huttmann, A. J. Martínez-Galera, V. Caciuc, N. Atodiresei, S. Schumacher, S. Standop, I. Hamada, T. O. Wehling, S. Blügel, T. Michely, *Phys. Rev. Lett.* **2015**, 115, 236101.

[11] A. Virkar, S. Mannsfeld, J. H. Oh, M. F. Toney, Y. H. Tan, G.-y. Liu, J. C. Scott, R. Miller, Z. Bao, *Adv. Funct. Mater*. **2009**, 19, 1962.

[12] N. N. Nguyen, H. C. Lee, M. S. Yoo, E. Lee, H. Lee, S. B. Lee, K. Cho, *Adv. Sci.* **2020**, 7, 1902315.

[13] S. Bommel, N. Kleppmann, C. Weber, H. Spranger, P. Schäfer, J. Novak, S. V. Roth, F. Schreiber, S. H. L. Klapp, S. Kowarik, *Nat. Commun*. **2014**, 5, 5388.
